# Intraoperative transcutaneous electrical acupoint stimulation combined with anesthesia to prevent postoperative cognitive dysfunction: A systematic review and meta-analysis

**DOI:** 10.1371/journal.pone.0313622

**Published:** 2025-01-09

**Authors:** Lin Gan, Kecheng Qian, Jinding Yang, Qian Cai, Qinyu Ye, Mengyuan Dai, Zhaoxing Jia, Tianxiang Jiang, Congcong Ma, Xianming Lin

**Affiliations:** The Third Clinical Medical College, Zhejiang Chinese Medical University, Key Laboratory of Acupuncture and Neurology of Zhejiang Province, Hangzhou, China; Sapienza University of Rome: Universita degli Studi di Roma La Sapienza, ITALY

## Abstract

**Background:**

Postoperative cognitive dysfunction (POCD) is associated with an increased risk of dementia and may lead to chronic neurodegeneration. The utilization of intraoperative Transcutaneous Electrical Acupoint Stimulation (TEAS) in conjunction with anesthesia is expected to become an effective preventive measure for POCD in clinical practice.

**Methods:**

We conducted a comprehensive literature review focusing on the use of TEAS in the prevention of POCD during surgical anesthesia. We searched various databases for relevant literature, including PubMed, Embase, Cochrane Library, Web of Science, China National Knowledge Infrastructure (CNKI), and Wanfang Data. The synthesis of data was performed using RevMan version 5.4.

**Results:**

Our meta-analysis incorporated data from 20 Randomized Controlled Trials (RCTs) involving 1549 patients. The findings revealed that intraoperative TEAS significantly reduced the incidence of POCD when compared to the control group [Odds Ratio (OR) 0.29, 95% Confidence Interval (CI) 0.22–0.39, *p* < 0.00001]. Moreover, patients receiving intraoperative TEAS exhibited a significant increase in MMSE scores (MD 1.21, 95% CI 0.53–1.89, *p* = 0.0005). Additionally, intraoperative TEAS demonstrated efficacy in reducing the contents of perioperative serum S100β protein (S100β), neuron-specific enolase (NSE), interleukin-6 (IL-6), and tumor necrosis factor-α (TNF-α) in patients, and the improvement of these indexes may be the potential mechanism of TEAS in preventing POCD.

**Conclusion:**

Our results suggest that intraoperative TEAS combined with anesthesia prevents cognitive dysfunction in the immediate postoperative period, however we need additional evidence of its utility in preventing long-term cognitive dysfunction. We advocate for the broader promotion and application of this approach in clinical surgical settings.

**Trial registration:**

PROSPERO (CRD42023457910).

## Introduction

Postoperative cognitive dysfunction (POCD) condition affects various facets of cognition including learning, memory, information processing, and overall cognitive function. It is prevalent following surgical procedures, with a higher incidence observed in the elderly population (≥60 years old) [1]. The condition is characterized by memory impairment, compromised information processing ability, and difficulties in concentration, accompanied by a range of adverse consequences including changes in mood and personality [2]. The current body of evidence suggests that the impact of pharmacological interventions on cognitive impairment remains uncertain, while non-pharmacological interventions are also considered effective preventive measures for POCD [3, 4]. Moreover, non-pharmacological interventions, especially transcutaneous electrical acupoint stimulation (TEAS) have emerged as a prominent area of research in recent years due to their cost-effectiveness, ease of implementation, and minimal occurrence of adverse reactions.

In recent years, transcutaneous electrical nerve stimulation (TENS) applied to acupuncture points has given rise to TEAS. Prior studies have highlighted the favorable effects of TEAS, including reduced intraoperative opioid use, alleviation of postoperative nausea and vomiting (PONV), pain relief, and improvement in postoperative cognitive function [5, 6]. In comparison to drug therapy, acupoint electrical stimulation offers the unique advantages of safety and fewer side effects [7, 8].

Although TEAS has been proved to have a good effect on improving cognition and memory, only a limited number of studies have comprehensively examined the efficacy and safety of TEAS combined with anesthesia during the operation for preventing POCD based on the Preferred Reporting Items for Systematic Reviews and Meta-analyses (PRISMA) guidelines. Therefore, we conducted a review of all currently available randomized controlled trials (RCTs) to assess the preventive effect of TEAS combined with anesthesia during the operation on POCD, aiming to provide evidence for clinical practice.

## Methods

We performed a systematic review and network meta-analysis according to the Preferred Reporting Items for Systematic Reviews and Meta-analyses (PRISMA) statement. In addition, this study has been registered with PROSPERO, with reference CRD42023457910.

### Ethics statement

The review protocol for this study was registered with PROSPERO on Sept 6, 2023 (CRD42023457910). All analyses were based on previous published studies; thus, no ethical approval or patient consent was required.

### Databases and search strategy

We conducted a comprehensive search of various databases, including PubMed, Embase, Cochrane Library, Web of Science, China National Knowledge Infrastructure (CNKI), and Wanfang Data. The search covered the inception dates of these databases until July 20, 2024, and no language restrictions were applied. Our search strategy involved the use of medical subject headings (MeSH) terms combined with free-text, encompassing terms such as ’Cognitive Function,’ ’Cognitive Dysfunction,’ ’Cognitive Impairments,’ ’POCD,’ ’Transcutaneous Electrical Acupoint Stimulation’, ’Acupoint Stimulation,’ and ’Electric Stimulation’, among others (see S1 Table).

### Selection criteria

This review prioritized RCTs due to their reduced susceptibility to confounding biases, including bias by indication [9]. The eligibility criteria for study selection were defined as follows: ① Participants aged 18 years and above who underwent surgery and anesthesia and exhibited normal cognitive function in preoperative assessments; ②The experiment group (EG) received TEAS; ③ The control group (CG) underwent a sham intervention or received no treatment; ④ The TEA intervention duration extended from 30 minutes before the operation to the conclusion of the operation; and ⑤ There were no restrictions on the type of operation. Exclusion criteria comprised studies with ① participants diagnosed with POCD; ② TEAS is used preoperatively or postoperatively; and ③ Articles utilizing the same datasets.

### Outcome measures

The primary outcomes of interest were the incidence of POCD and cognitive function scores, assessed using the Mini-Mental State Examination (MMSE). Secondary outcomes encompassed postoperative pain, adverse reactions, and length of hospital stay. Additionally, we explored secondary outcomes related to levels of inflammatory factors associated with cognitive dysfunction, including perioperative serum S100β protein (S100β), Interleukin-6 (IL-6), neuron specific enolase (NSE), and tumor necrosis factor-α (TNF-α).

### Data extraction and quality assessment

Two investigators (GL and QKC) independently gathered data from eligible studies and entered the outcome data into a pre-designed spreadsheet. Any discrepancies during the crosschecking process were resolved through discussion. In cases where consensus could not be reached, a third investigator (YJD) served as an arbitrator. The key information extracted from the included articles encompassed study design, studied populations, type of operation, type of anesthesia, intervention details, outcomes, and postoperative test time. The Cochrane risk of bias tool [10] was utilized to assess the methodological quality and risks of bias in individual studies. The certainty of the evidence for each outcome was evaluated using the Grading of Recommendations, Assessment, Development, and Evaluations (GRADE) framework [11].

### Data synthesis and analysis

The synthesis of data was performed using RevMan version 5.4. For dichotomous outcomes, such as the incidence of POCD, we computed the odds ratio (OR) along with the corresponding 95% confidence interval (CI). Regarding continuous outcomes, such as MMSE scores, we aggregated the mean difference (MD) and its 95% CI. An *I*^*2*^ value of less than 50% indicates that no heterogeneity was observed and a fixed effects model was used. Statistical significance was defined as *p* < 0.05. The studies included in this paper did not have any missing data.

### Subgroup analyses and investigation of heterogeneity

When a substantial number of studies provided pertinent characteristics, we conducted subgroup analyses for assessments at different time points. Heterogeneity among the studies was evaluated using Cochran Q tests (χ2 tests for heterogeneity), considering a Q test with p < 0.10 or *I*^*2*^ > 50% as indicative of significant statistical heterogeneity. The random-effects model was employed to compute the effect size, accommodating variations between the studies [12].

### Publication bias

We assessed publication bias in cases where a sufficient number of studies were available (n ≥ 10). Asymmetric funnel plots and Harbord tests were employed to evaluate potential publication bias when the OR was used as an effect estimate, and the results indicated no significant heterogeneity between the studies. Additionally, when the mean difference (MD) served as an effect estimate, we utilized asymmetric funnel plots and Egger’s test to investigate potential publication bias [13]. To identify and rectify funnel plot asymmetries arising from publication bias, we applied the trim-and-fill method [14].

## Results

### Study selection and characteristics

**Fig 1:**
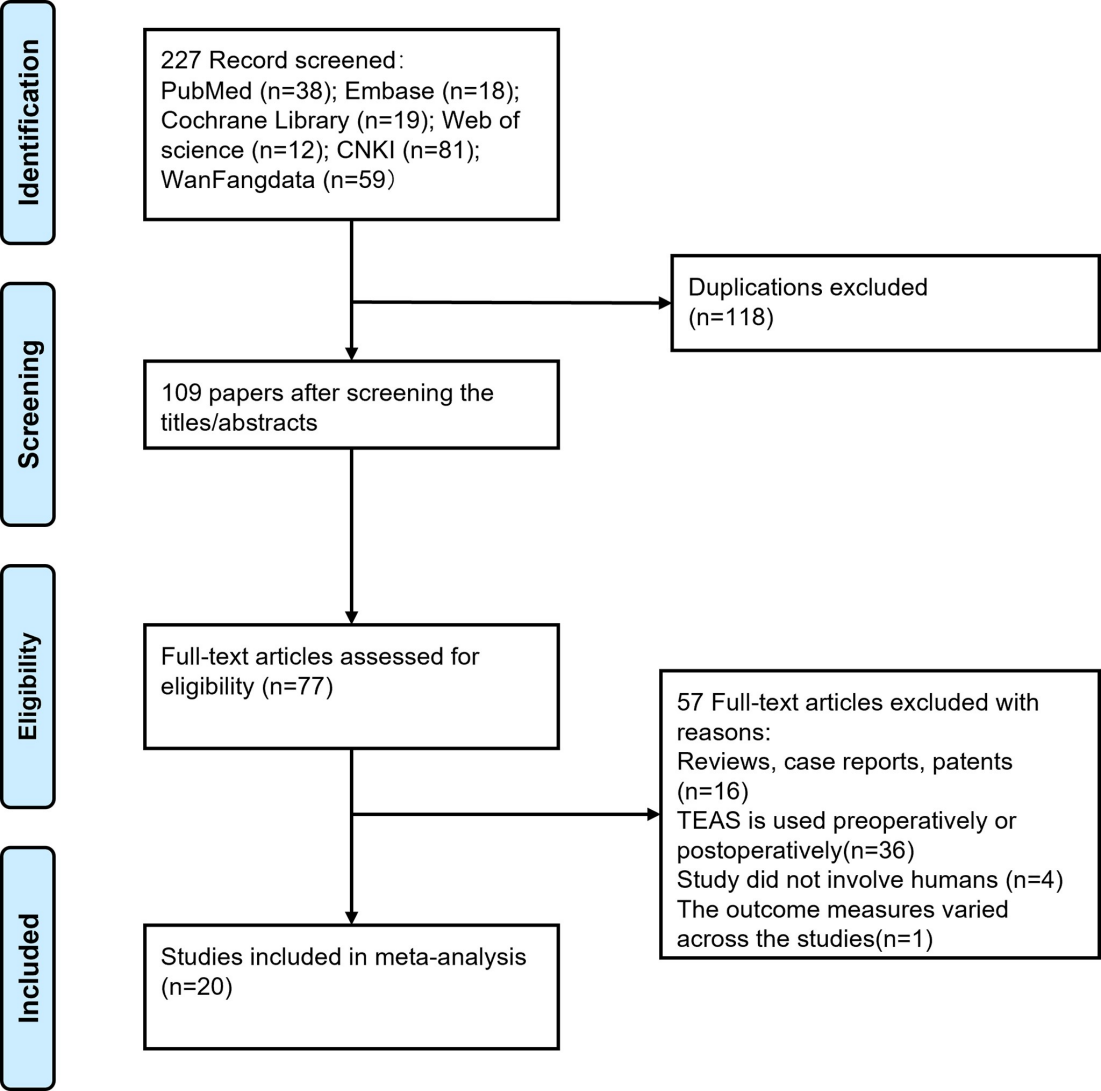
The flow chart of literature screening.

The database search yielded a total of 227 articles. Following adherence to the inclusion and exclusion criteria, 20 RCTs involving 1,549 participants were deemed eligible for data extraction. The screening process is visually represented in **Fig 1**. All trials included in this review were conducted in China and published in both Chinese and English. Among the included trials, 15 had participants with an average age ranging over 65 years. Of these 20 reported interventions, the duration of the TEAS intervention was from 30min before anesthesia and conducted until the end of surgery. The evaluation time points in the included studies ranged from 0 to 7 days after surgery (shown in **Table 1**).

**Table 1:** The characteristics of included studies.

First author	Age (years) EG/CG	Gender Male/Female	BMI (kg/m^2^) EG/CG	Sample size EG/CG	Type of operation/anesthesia	Intervention			Primary Outcomes	Secondary Outcomes	Postoperative test time
						EG	CG	Acupoints/Stimulation frequency/time			
Cai 2021 [15]	68.5 ± 4.6/69.7 ± 4.8	45/41	/	43/43	hip replacement/general anesthesia	TEAS	No treatment	GV20, PC6, ST36, SP6; 2/100Hz; at 30 min before the induction of anesthesia, and conducted until the end of operation.	MMSE, POCD Incidence	NSE, S-100β, IL-1β, IL-6 and TNF-α	1, 3, 5d
Chen 2022 [16]	70.82 ± 7.19/71.86 ± 7.24	69/40	/	56/53	single whole thoracoscopic lobectomy/general anesthesia	TEAS	Sham TEAS	EX-HN5, GB20; 2/100Hz; at 30 min before the induction of anesthesia, and conducted until the end of operation.	MMSE	NES, S100β and p-Tau	24, 72h
Guo 2023 [17]	70.09 ± 3.95/70.56 ± 4.59	47/63	24.09 ± 2.78/23.63 ± 2.85	55/55	thoracoscopic pulmonary resection/general anesthesia	TEAS	Sham TEAS	GV20, PC6, LI4, ST36; 2/100Hz; at 30 min before the induction of anesthesia, and conducted until the end of operation.	POCD Incidence, MoCA	MMSE, NRS, EORTC-QLQ-C30	1, 7, 30d
Li 2016 [18]	65.7 ± 6.12/66.5 ± 3.95	34/26	/	30/30	radical thoracoscopic lung cancer operation/general anesthesia	TEAS	No treatment	PC6, ST36; 2/100Hz; at 30 min before the induction of anesthesia, and conducted until the end of operation.	MMSE, POCD Incidence	NSE, S100β	1, 3d
Lin 2013 [19]	68.5 ± 2.8/67.3 ± 2.7	34/15	/	25/24	resection of gastrointestinal tumors/general anesthesia	TEAS	No treatment	GV20, EX-HN5, PC6; 2/100Hz; at 30 min before the induction of anesthesia, and conducted until the end of operation.	MMSE, POCD Incidence	S100β	3d
Liu 2021 [20]	70.80 ± 5.41/69.68 ± 4.85	51/49	24.75 ± 2.57/24.82 ± 3.47	50/50	laparoscopic radical resection of colon cancer/general anesthesia	TEAS	No treatment	PC6, LI4, ST36; 2/100Hz; from 30 min before the induction of anesthesia to the end of surgery.	IL-6, hs-CRP and CGRP	POCD Incidence	T1: 1 h after the beginning of surgery; T2: the end of surgery.
Lu 2019 [21]	72.07 ± 2.53/71.29 ± 2.31	46/45	21.88 ± 0.42/21.95 ± 0.38	46/45	hip replacement/general anesthesia	TEAS	No treatment	GV20, PC6, GB20; 2/100Hz; at 30 min before the induction of anesthesia, and conducted until the end of operation.	MMSE, POCD Incidence	/	72h
Mi 2018 [22]	44 ± 6/45 ± 8	54/46	/	50/50	laparoscopic cholecystectomy/general anesthesia	TEAS	No treatment	LI4, PC6, ST36; 2/100Hz; from 30 min before the induction of anesthesia to the end of surgery.	QoR-40, MMSE	/	4,8, 24,48h
Ni 2009 [23]	20–67	24/26	/	25/25	craniotomy/general anesthesia	TEAS	No treatment	LI4, LI11, ST36, SP6; 2/100Hz; from 30 min before the induction of anesthesia to the end of surgery.	SOD, MDA, S100β	MMSE	1, 24, 48h
Ni 2015 [24]	68.7 ± 5.3/69.2 ± 5.0	38/22	/	30/30	laparoscopic resection of rectal cancer/general anesthesia	TEAS	No treatment	GV20, PC6, ST36, SP6; 2/100Hz; at 30 min before the induction of anesthesia, and conducted until the end of operation.	MMSE, POCD Incidence	SOD, MDA, S100β	1, 3, 5, 7d
Tang 2016 [25]	69.6 ± 5.8/70.1 ± 6.3	49/41	/	45/45	colorectal cancer surgery/general anesthesia	TEAS	No treatment	GV20, DU24; 2/100Hz; at 30 min before the induction of anesthesia, and conducted until the end of operation.	MMSE, POCD Incidence	/	1, 3, 5, 7d
Tang 2020 [26]	50.17 ± 4.82/49.82 ± 4.19	48/42	23.96 ± 0.83/23.84 ± 0.76	45/45	laparoscopic radical resection of colorectal cancer/general anesthesia	TEAS	No treatment	LI4, ST36, PC6; at 30 min before the induction of anesthesia, and conducted until the end of operation.	MMSE, POCD Incidence, VAS	IL-6, IL-1, TNF-α	1, 3, 5, 7d
Wang 2016 [27]	70.3 ± 4.2/69.5 ± 4.4	34/26	/	30/30	abdominal operation/ general anesthesia	TEAS	No treatment	GV20, ST36, PC6, SP6; at 30 min before the induction of anesthesia, and conducted until the end of operation.	MMSE, POCD Incidence	/	1, 3, 7d
Wang 2022 [28]	66.3 ± 6.5/65.4 ± 7.6	71/49	/	60/60	hip fracture surgery/general anesthesia	TEAS	Sham TEAS	GV20, PC6, GV14; 2/100Hz; at 30 min before the induction of anesthesia, and conducted until the end of operation.	MMSE, POCD Incidence	IL-6, IL-1β, NSE, S100β	3, 7d
Wang 2023 [29]	70.2 ± 4.2/72.3 ± 5.4	22/18	64.36 ± 9.08 64.13 ± 9.21	20/20	total knee arthroplasty/lumbar and epidural anesthesia	TEAS	No treatment	ST36, SP6; at 30 min before the induction of anesthesia, and conducted until the end of operation.	MMSE, POCD Incidence	IL-6, IL-1, S100β, TNF-α	1, 3, 7d
Wei 2016 [30]	55.2 ± 6.14/56.5 ± 4.59	0/40	/	20/20	electrical gynecological laparoscopic surgery/ general anesthesia	TEAS	No treatment	PC6, GV20, GB20; 2/100Hz; at 30 min before the induction of anesthesia, and conducted until the end of operation.	MMSE, POCD Incidence	NES, S100β	1, 3d
Wei 2022 [31]	72.32 ± 10.21/72.14 ± 11.34	46/44	23.63 ± 4.01/22.37 ± 5.74	46/44	video-assisted thoracoscopic surgical (VATS) lobectomy/general anesthesia	TEAS	Sham TEAS	PC6, ST36; 2/10 Hz; at 30 min before the induction of anesthesia, and conducted until the end of operation.	POCD Incidence, MMSE, MoCA	S100β, NSE	1, 3, 5, 7d
Wu 2019 [32]	72.32 ± 5.29/71.89 ± 5.11	32/52	/	42/42	cardiac surgery/general anesthesia	TEAS	No treatment	PC6, ST36, SP6; 2/100Hz; at 30 min before the induction of anesthesia, and conducted until the end of operation.	MMSE	COR, β-EP, VAS	72h
Yang 2015 [33]	65–80	0/60	/	30/30	gynecological laparoscopic surgery/general anesthesia	TEAS	Sham TEAS	PC6, ST36; 2/100Hz; at 30 min before the induction of anesthesia, and conducted until the end of operation.	MMSE, POCD Incidence	IL-6, S100β	1, 3, 5d
Zhu 2016 [34]	<60	0/60	/	30/30	laparoscopic surgery/general anesthesia	TEAS	No treatment	ST36, PC6; at 30 min before the induction of anesthesia, and conducted until the end of operation.	MMSE, POCD Incidence	S100β	24h

MoCA: Montreal cognitive assessment, QoR-40: a 40-item questionnaire that measures the quality of recovery after surgery and anesthesia, SOD: superoxide dismutase, MDA: malondialdehyde

### Quality assessments

**Fig 2:**
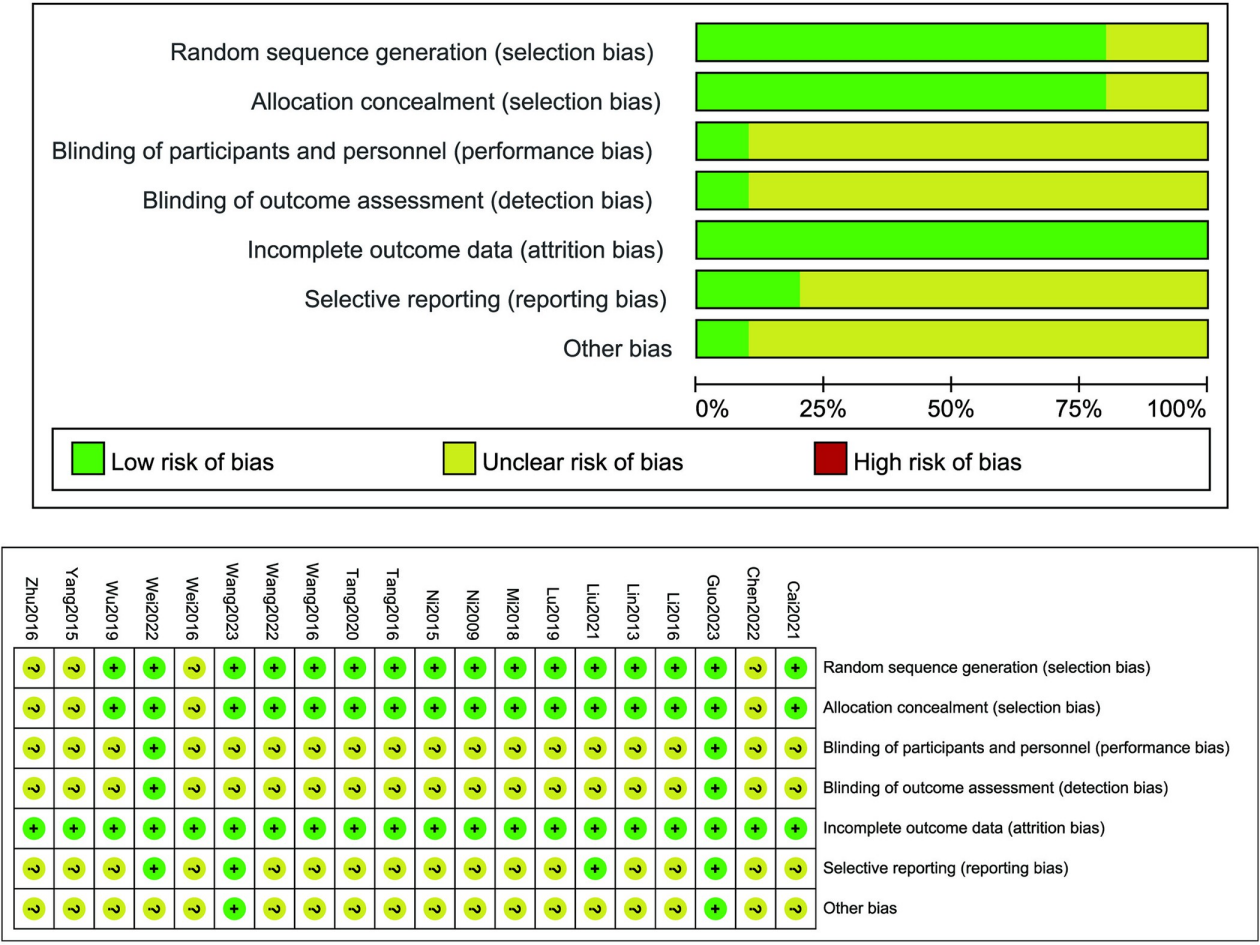
Risk of bias summary of the studies.

All studies included in this review were identified as randomized. Of these, 14 studies (70%) utilized a random number table for randomization, while 5 studies were randomized but did not describe specific randomization procedure. Sixteen studies (80%) provided a description of the proper method for achieving allocation concealment. Eighteen studies lacked detailed descriptions of the blinding approach to participants and personnel. No study demonstrated high risk regarding incomplete outcome data. The data in this paper are real and reliable. The methodological quality of the included trials is depicted in **Fig 2**.

### Primary outcomes

#### Incidence of POCD

**Fig 3:**
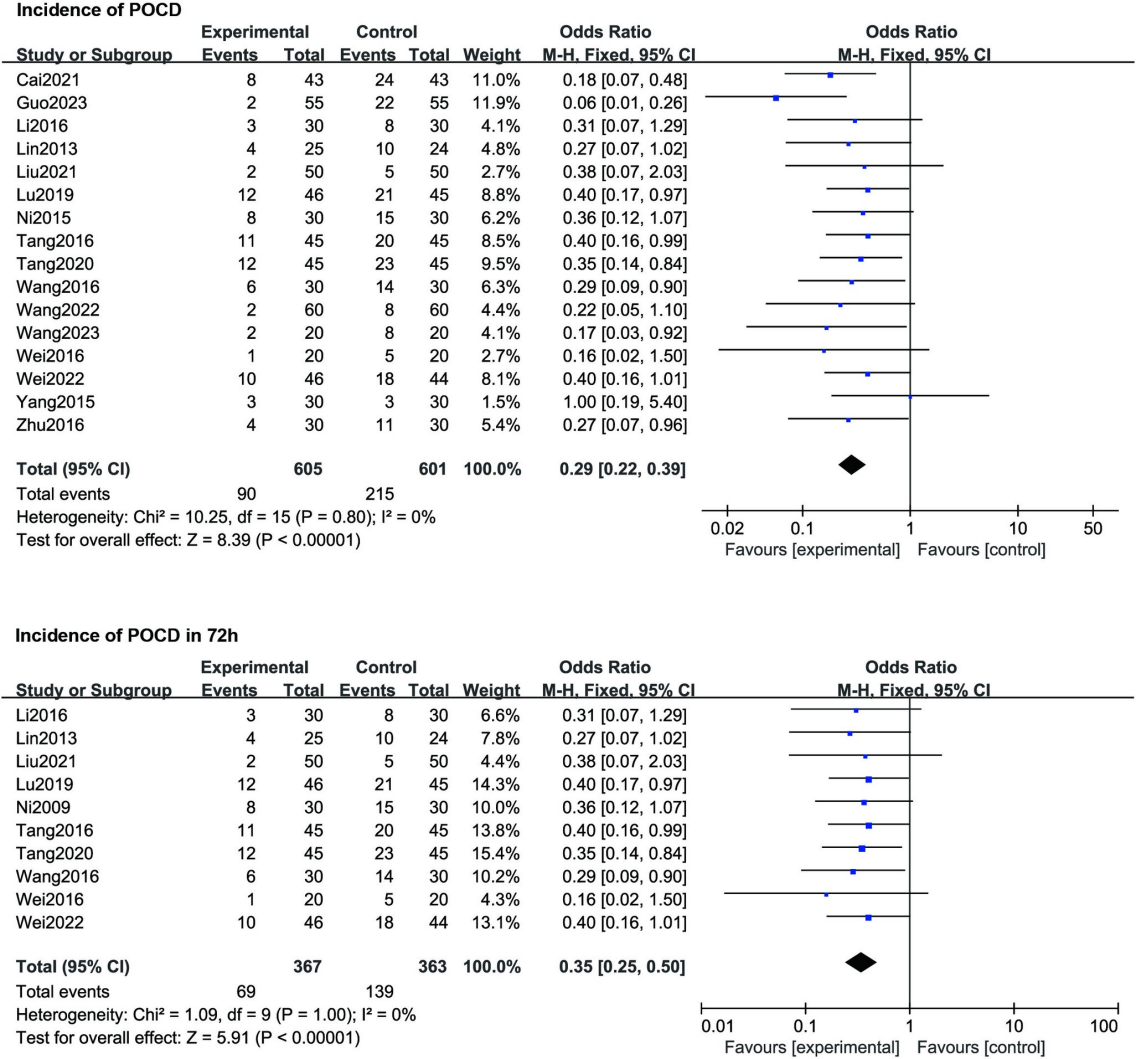
Forest plot compares the incidence of POCD and the incidence of POCD in 72h between TEAS and control treatment.

Sixteen studies provided data on the incidence of POCD. A statistical analysis was conducted using the last recorded data from these studies. Overall, the results indicated that, in comparison to the Control group, the TEAS group exhibited a significantly lower incidence of POCD (OR 0.29, 95% CI 0.22–0.39, *p* < 0.00001; shown in **Fig 3, top**). We did not find any obvious publication bias in the included studies using funnel plots (S1 Fig). The time required to calculate the incidence of POCD varies from 1 to 30 days, as indicated in **Table 1**, rendering it incomparable. Hence, we also considered the incidence of POCD within 72 hours after surgery for statistical analysis, with this outcome being recorded in 10 studies. The findings demonstrated that the combination of TEAS and anesthesia effectively reduced the occurrence of POCD within 72 hours post-surgery (OR 0.33, 95% CI 0.25–0.50, *p* < 0.00001; shown in **Fig 3, bottom**).

#### MMSE scores

**Fig 4:**
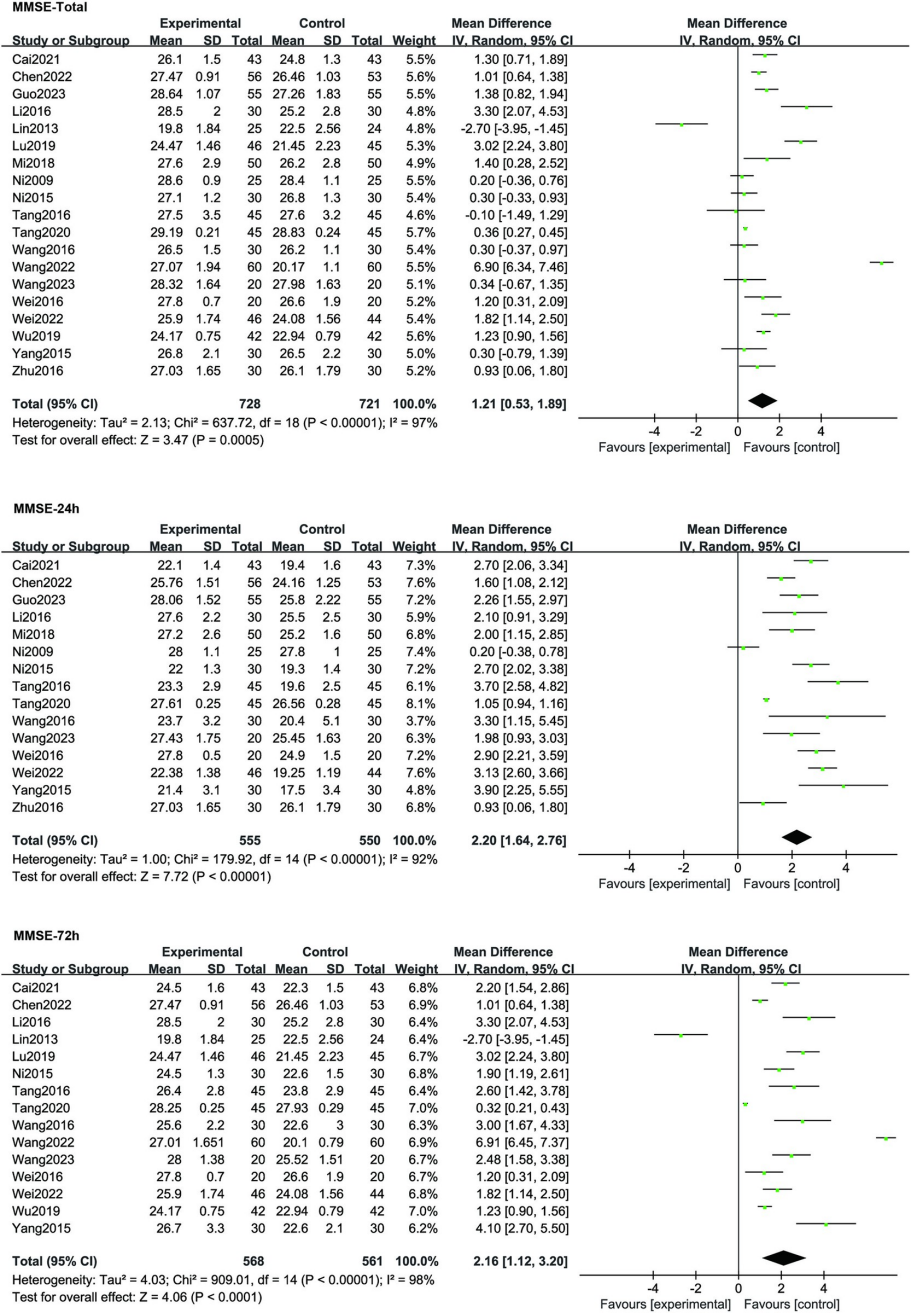
Forest plot compares the MMSE scores between TEAS and control treatment.

19 studies reported MMSE scores. The meta-analysis of the final assessments indicated a significant improvement in MMSE scores in the TEAS group compared to the Control group (MD 1.21, 95% CI 0.53–1.89, *p* = 0.0005). Further analysis at specific time points revealed that, at postoperative 24 hours, the TEAS group exhibited significantly enhanced MMSE scores compared to the Control group (MD2.20, 95% CI 1.64–2.76, *p* <0.00001). This trend persisted at 72h postop mentioned in fourteen studies, with a notable improvement in MMSE scores in the TEAS group compared to the Control group (MD 2.16, 95% CI 1.12–3.20, *p* <0.00001; shown in **Fig 4**).

### Secondary outcomes

**Fig 5:**
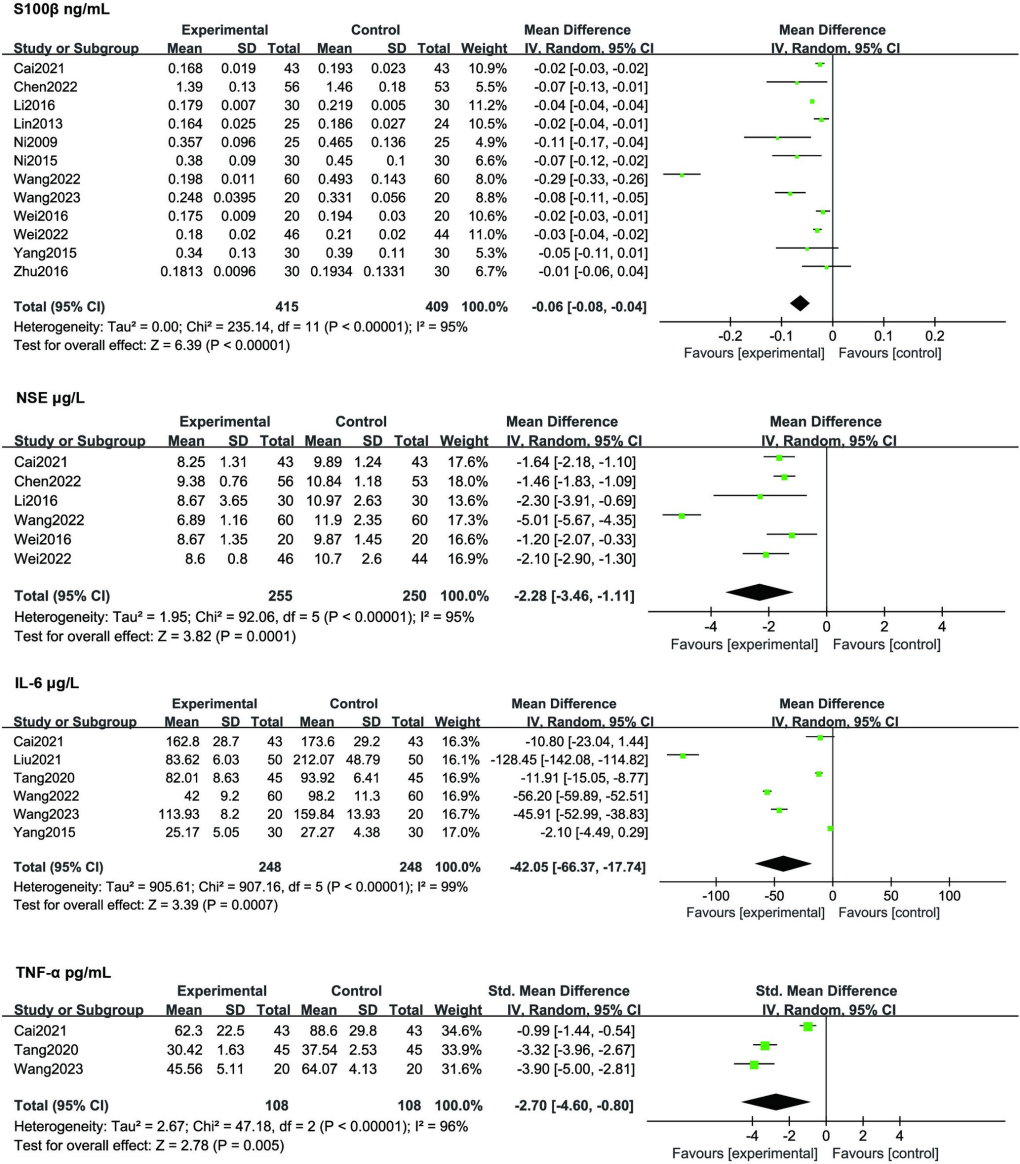
Forest plot compares the inflammatory factors between TEAS and control treatment.

We primarily focused on secondary outcome measures related to inflammatory factors associated with cognitive dysfunction development. The included articles investigated four types of inflammatory factors, including S100β, IL-6, NES, and TNF-α shown in **Fig 5**. The level of inflammatory markers 1 day after surgery was chosen as the standardized time point for statistical analysis, as it aligns with the majority of studies in terms of measurement timing. The meta-analysis revealed a significantly lower S100β content in the TEAS group compared to the Control group in 12 studies (MD -0.06, 95% CI -0.08- -0.04, *p* <0.00001). Similarly, the meta-analysis indicated a significantly lower NSE content in the TEAS group compared to the Control group in 6 studies (MD -2.28, 95% CI -3.46- -1.11, *p* = 0.00001). Additionally, there was a lower IL-6 content in the TEAS group compared to the Control group in 6 studies (MD -42.05, 95% CI -66.37- -17.74, *p* = 0.0007), and lower TNF-α content in the TEAS group compared to the Control group in 3 studies (MD -2.70, 95% CI -4.60- -0.80, *p* = 0.005)

## Discussion

In this study, we investigated the prophylactic effect of intraoperative TEAS combined with anesthesia on postoperative cognitive changes. Our findings demonstrated a significant improvement in MMSE scores and a reduction in the incidence of POCD at 24- and 72- hours following surgery. These consistent results strongly support the conclusion that TEAS effectively prevents POCD in surgical patients. Furthermore, our meta-analysis revealed that intraoperative TEAS combined with anesthesia significantly decreases postoperative levels of IL-6, NSE, and S100β proteins in blood samples. These findings suggest potential mechanisms through which TEAS ameliorates cognitive dysfunction in surgical patients.

Currently, the definition and the standardization of evaluation methods of POCD remain ambiguous. The term POCD typically encompasses transient or persistent cognitive impairment following anesthesia or surgical procedures in the majority of previous research studies [1]. In 2018, an internationally recognized renaming proposal [35] recommends that ‘perioperative neurocognitive disorders, PND’ be used as an overarching term for cognitive impairment identified in the preoperative or postoperative period. Postoperative delirium (POD) refers to neurocognitive dysfunction within 7 days after surgery, while POCD can be considered when the impairment persists beyond one week, particularly up to six months late [36]. The majority of the included studies, however, have not adequately assessed the long-term or short-term cognitive function of patients after surgery, which poses a challenge in distinguishing between POD and POCD based on the proposed renaming. The expectation is that forthcoming clinical trials will be able to effectively differentiate between the concepts of POD and POCD, thereby yielding more precise outcomes.

Neuroinflammation is a common pathological feature and onset in multiple cognitive disorders, including POCD [37]. The central nervous system and peripheral tissues can both experience immunoinflammation and heightened cytokine release, which may be triggered by factors such as anesthesia, surgery, and trauma [38, 39]. A clinical study by *Lin et al*. [40] has confirmed that a significant increase in serum IL-6 levels may pose a potential risk for cognitive dysfunction in the elderly. The study conducted by *Xie et al*. [41] unveiled that alterations in NSE and S100β had a significant impact on the occurrence of POCD. Studies emphasize the pivotal roles of IL-6, NSE, TNF-α and S100β proteins in the domains of learning, memory, and cognitive function, wherein heightened expression is associated with cognitive dysfunction as inflammatory indicators [42–44]. Our study has confirmed that the use of TEAS to inhibit these proteins, which are likely to be involved in the treatment of POCD, can enhance the potential mechanism and guide future research directions.

The brain-protective effects of electroacupuncture (EA) have been substantiated by clinical trials, as it effectively inhibits inflammatory responses and regulates pro-inflammatory and anti-inflammatory factors. Consequently, this intervention proves to be an effective preventive or therapeutic measure in reducing POCD among elderly patients [45, 46]. The TEAS, by addressing the operational challenges and discomfort associated with EA, offers a non-invasive and straightforward administration method that is highly accepted by patients and generally free from adverse reactions [47]. The efficacy of TEAS has been demonstrated in the treatment of various conditions associated with learning and memory impairment, including dementia [48, 49] and cerebral stroke [50]. Previous studies have consistently confirmed TEAS’s effectiveness in treating cognitive, emotional, and encephalopathy-related disorders [47, 51]. Previous meta-analyses have also reported a favorable impact of TEAS treatment on the prevention of POCD [52–54], in comparison to the updated RCT data included in this study. Moreover, this study precisely limited the intervention time point of TEAS and provided clear information regarding the treatment duration and frequency in the treatment group, enhancing accuracy.

The available evidence suggests that both TEAS and electricity exert similar regulatory effects on the same brain functional area, with no statistically significant difference observed between TEAS and acupuncture at the identical acupoint [55]. The selection of acupuncture points is a critical parameter, and there are variations in TEAS acupoint combinations across different studies. In this review of acupoint selections, Neiguan (PC6) was the most frequently mentioned acupoint (16 studies), followed by Zusanli (ST36, 14 studies), Baihui (GV20, 9 studies), Hegu (LI6, 5 studies), and Sanyinjiao (SP6, 6 studies). *Lin et al*. [56] demonstrated that the intervention of various acupoints, including Baihui (GV20) and Neiguan (PC6), effectively ameliorates cognitive impairment symptoms in elderly patients. These acupoints are deeply rooted in traditional Chinese medicine theory and renowned for their regulatory effects on the mind. Moreover, their larger exposed surface area facilitates percutaneous acupuncture point stimulation, which likely contributes to their frequent selection. Nevertheless, we believe that future research should focus on exploring the exciting realm of TEAS and optimizing stimulation parameters input to identify the most suitable points for achieving both effectiveness and safety, thereby enhancing overall outcomes.

### Limitations

Several limitations of this study merit discussion. Firstly, the limited number of original studies is a drawback of this study. Secondly, the literature included in our study did not encompass all indicators pertaining to POCD, restricting the comprehensiveness of our findings. Additionally, the duration of surgery and the class of narcotic drugs can influence the occurrence of POCD. However, these factors were not extensively detailed in the included studies, precluding a subgroup analysis for these variables.

## Conclusion

Our meta-analysis demonstrates that intraoperative TEAS combined with anesthesia prevents cognitive dysfunction in the immediate postoperative period, however we need additional evidence of its utility in preventing long-term cognitive dysfunction. We recommend the wider promotion and implementation of TEAS combined with anesthesia in order to effectively prevent POCD in the clinical surgical setting.

## Supporting information

S1 Checklist PRISMA checklist. PRISMA statement for reporting systematic reviews and meta-analyses.
(DOCX)

S1 Raw data Raw data extraction. (DOCX)

S1 Fig Funnel plot of incidence of POCD studies. (TIF)

S2 Fig Funnel plot of other outcomes. (TIF)

S1 Table Search strategy. (DOCX)

S2 Table All studies identified in the literature search. (DOCX)
