# Supplementary material for: Intraoperative transcutaneous electrical acupoint stimulation combined with anesthesia to prevent postoperative cognitive dysfunction: A systematic review and meta-analysis
Source: PLoS One. 2025 Jan 9;20(1):e0313622. doi: 10.1371/journal.pone.0313622 (PMC11717303; doi:10.1371/journal.pone.0313622)
Supplement: S1 Raw data — (DOCX) [file pone.0313622.s002.docx]

**Raw Data extraction.**

| **Incidence of POCD** |  |  |  |  |  |  |
| --- | --- | --- | --- | --- | --- | --- |
| study | EG |  | CG |  |  |  |
|  | n1 | total | n1 | total |  | Time |
| Cai2021 | 8 | 43 | 24 | 43 |  | 5d |
| Guo2023 | 2 | 55 | 22 | 55 |  | 30d |
| Li2016 | 3 | 30 | 8 | 30 |  | 72h |
| Lin2013 | 4 | 25 | 10 | 24 |  | 72h |
| Liu2021 | 2 | 50 | 5 | 50 |  | 72h |
| Lu2019 | 12 | 46 | 21 | 45 |  | 72h |
| Ni2015 | 8 | 30 | 15 | 30 |  | 72h |
| Tang2016 | 11 | 45 | 20 | 45 |  | 72h |
| Tang2020 | 12 | 45 | 23 | 45 |  | 72h |
| Wang2016 | 6 | 30 | 14 | 30 |  | 72h |
| Wang2023 | 2 | 20 | 8 | 20 |  | 7d |
| Wang2022 | 2 | 60 | 8 | 60 |  | 7d |
| Wei2016 | 1 | 20 | 5 | 20 |  | 72h |
| Wei2022 | 10 | 46 | 18 | 44 |  | 72h |
| Yang2015 | 3 | 30 | 3 | 30 |  | 5d |
| Zhu2016 | 4 | 30 | 11 | 30 |  | 1d |
|  |  |  |  |  |  |  |
| **Incidence of POCD in 72h** |  |  |  |  |  |  |
| study |  |  |  |  |  |  |
|  | n1 | total | n1 | total |  |  |
| Li2016 | 3 | 30 | 8 | 30 |  | 72h |
| Lin2013 | 4 | 25 | 10 | 24 |  | 72h |
| Liu2021 | 2 | 50 | 5 | 50 |  | 72h |
| Lu2019 | 12 | 46 | 21 | 45 |  | 72h |
| Ni2015 | 8 | 30 | 15 | 30 |  | 72h |
| Tang2016 | 11 | 45 | 20 | 45 |  | 72h |
| Tang2020 | 12 | 45 | 23 | 45 |  | 72h |
| Wang2016 | 6 | 30 | 14 | 30 |  | 72h |
| Wei2016 | 1 | 20 | 5 | 20 |  | 72h |
| Wei2022 | 10 | 46 | 18 | 44 |  | 72h |

| **MMSE** |  |  |  |  |  |  |  |
| --- | --- | --- | --- | --- | --- | --- | --- |
| study | EG |  |  | CG |  |  |  |
|  | mean1 | sd1 | n1 | mean2 | sd2 | n2 | Time |
| Wei2022 | 25.9 | 1.74 | 46 | 24.08 | 1.56 | 44 | 72h/24h/5d/7d |
| Chen2022 | 27.47 | 0.91 | 56 | 26.46 | 1.03 | 53 | 72h/24h |
| Lin2013 | 19.8 | 1.84 | 25 | 22.5 | 2.56 | 24 | 72h |
| Mi2018 | 27.6 | 2.9 | 50 | 26.2 | 2.8 | 50 | 24h/48h |
| Ni2009 | 28.6 | 0.9 | 25 | 28.4 | 1.1 | 25 | 24h/48h |
| Guo2023 | 28.64 | 1.07 | 55 | 27.26 | 1.83 | 55 | 1, 7, 30d |
| Wei2016 | 27.8 | 0.7 | 20 | 26.6 | 1.9 | 20 | 24/72h |
| Ni2015 | 27.1 | 1.2 | 30 | 26.8 | 1.3 | 30 | 1,3,5,7d |
| Tang2016 | 27.5 | 3.5 | 45 | 27.6 | 3.2 | 45 | 1,3,5,7d |
| Wang2022 | 27.07 | 1.94 | 60 | 20.17 | 1.1 | 60 | 3,7d |
| Lu2019 | 24.47 | 1.46 | 46 | 21.45 | 2.23 | 45 | 72h |
| Li2016 | 28.5 | 2 | 30 | 25.2 | 2.8 | 30 | 24/72h |
| Wu2019 | 24.17 | 0.75 | 42 | 22.94 | 0.79 | 42 | 72h |
| Cai2021 | 26.1 | 1.5 | 43 | 24.8 | 1.3 | 43 | 1,3,5d |
| Yang2015 | 26.8 | 2.1 | 30 | 26.5 | 2.2 | 30 | 1,3,5d |
| Tang2020 | 29.19 | 0.21 | 45 | 28.83 | 0.24 | 45 | 1,3,5,7d |
| Zhu2016 | 27.03 | 1.65 | 30 | 26.1 | 1.79 | 30 | 24h |
| Wang2016 | 26.5 | 1.5 | 30 | 26.2 | 1.1 | 30 | 1,3,7d |
| Wang2023 | 28.32 | 1.64 | 20 | 27.98 | 1.63 | 20 | 1,3,7d |
|  |  |  |  |  |  |  |  |
| **24hMMSE** |  |  |  |  |  |  |  |
| Wei2022 | 22.38 | 1.38 | 46 | 19.25 | 1.19 | 44 |  |
| Chen2022 | 25.76 | 1.51 | 56 | 24.16 | 1.25 | 53 |  |
| Mi2018 | 27.2 | 2.6 | 50 | 25.2 | 1.6 | 50 |  |
| Ni2009 | 28 | 1.1 | 25 | 27.8 | 1.0 | 25 |  |
| Guo2023 | 28.06 | 1.52 | 55 | 25.8 | 2.22 | 55 |  |
| Wei2016 | 27.8 | 0.5 | 20 | 24.9 | 1.5 | 20 |  |
| Ni2015 | 22 | 1.3 | 30 | 19.3 | 1.4 | 30 |  |
| Tang2016 | 23.3 | 2.9 | 45 | 19.6 | 2.5 | 45 |  |
| Li2016 | 27.6 | 2.2 | 30 | 25.5 | 2.5 | 30 |  |
| Cai2021 | 22.1 | 1.4 | 43 | 19.4 | 1.6 | 43 |  |
| Yang2015 | 21.4 | 3.1 | 30 | 17.5 | 3.4 | 30 |  |
| Tang2020 | 27.61 | 0.25 | 45 | 26.56 | 0.28 | 45 |  |
| Zhu2016 | 27.03 | 1.65 | 30 | 26.1 | 1.79 | 30 |  |
| Wang2016 | 23.7 | 3.2 | 30 | 20.4 | 5.1 | 30 |  |
| Wang2023 | 27.43 | 1.75 | 20 | 25.45 | 1.63 | 20 |  |
|  |  |  |  |  |  |  |  |
| **72hMMSE** |  |  |  |  |  |  |  |
| Wei2022 | 25.9 | 1.74 | 46 | 24.08 | 1.56 | 44 |  |
| Chen2022 | 27.47 | 0.91 | 56 | 26.46 | 1.03 | 53 |  |
| Lin2013 | 19.8 | 1.84 | 25 | 22.5 | 2.56 | 24 |  |
| Wei2016 | 27.8 | 0.7 | 20 | 26.6 | 1.9 | 20 |  |
| Ni2015 | 24.5 | 1.3 | 30 | 22.6 | 1.5 | 30 |  |
| Tang2016 | 26.4 | 2.8 | 45 | 23.8 | 2.9 | 45 |  |
| Wang2022 | 27.01 | 1.651 | 60 | 20.1 | 0.79 | 60 |  |
| Lu2019 | 24.47 | 1.46 | 46 | 21.45 | 2.23 | 45 |  |
| Li2016 | 28.5 | 2 | 30 | 25.2 | 2.8 | 30 |  |
| Wu2019 | 24.17 | 0.75 | 42 | 22.94 | 0.79 | 42 |  |
| Cai2021 | 24.5 | 1.6 | 43 | 22.3 | 1.5 | 43 |  |
| Yang2015 | 26.7 | 3.3 | 30 | 22.6 | 2.1 | 30 |  |
| Tang2020 | 28.25 | 0.25 | 45 | 27.93 | 0.29 | 45 |  |
| Wang2016 | 25.6 | 2.2 | 30 | 22.6 | 3 | 30 |  |
| Wang2023 | 28 | 1.38 | 20 | 25.52 | 1.51 | 20 |  |

| **24hS100β** |  |  |  |  |  |  |
| --- | --- | --- | --- | --- | --- | --- |
| study | EG |  |  | CG |  |  |
|  | mean1 | sd1 | n1 | mean2 | sd2 | n2 |
| Ni2009 | 0.357 | 0.096 | 25 | 0.465 | 0.136 | 25 |
| Wei2022 | 0.18 | 0.02 | 46 | 0.21 | 0.02 | 44 |
| Chen2022 | 1.39 | 0.13 | 56 | 1.46 | 0.18 | 53 |
| Lin2013 | 0.164 | 0.025 | 25 | 0.186 | 0.027 | 24 |
| Wei2016 | 0.175 | 0.009 | 20 | 0.194 | 0.03 | 20 |
| Ni2015 | 0.38 | 0.09 | 30 | 0.45 | 0.1 | 30 |
| WangSG2022 | 0.198 | 0.011 | 60 | 0.493 | 0.143 | 60 |
| Li2016 | 0.179 | 0.007 | 30 | 0.219 | 0.005 | 30 |
| Cai2021 | 0.168 | 0.019 | 43 | 0.193 | 0.023 | 43 |
| Yang2015 | 0.34 | 0.13 | 30 | 0.39 | 0.11 | 30 |
| Zhu2016 | 0.1813 | 0.0096 | 30 | 0.1934 | 0.1331 | 30 |
| Wang2023 | 0.248 | 0.0395 | 20 | 0.331 | 0.056 | 20 |
|  |  |  |  |  |  |  |
| **IL-6** |  |  |  |  |  |  |
| study |  |  |  |  |  |  |
|  | mean1 | sd1 | n1 | mean2 | sd2 | n2 |
| Liu2021 | 83.62 | 6.03 | 50 | 212.07 | 48.79 | 50 |
| WangSG2022 | 42 | 9.2 | 60 | 98.2 | 11.3 | 60 |
| Cai2021 | 162.8 | 28.7 | 43 | 173.6 | 29.2 | 43 |
| Yang2015 | 25.17 | 5.05 | 30 | 27.27 | 4.38 | 30 |
| Tang2020 | 82.01 | 8.63 | 45 | 93.92 | 6.41 | 45 |
| Wang2023 | 113.93 | 8.2 | 20 | 159.84 | 13.93 | 20 |
|  |  |  |  |  |  |  |
| **NSE** |  |  |  |  |  |  |
| study |  |  |  |  |  |  |
|  | mean1 | sd1 | n1 | mean2 | sd2 | n2 |
| Wei2022 | 8.6 | 0.8 | 46 | 10.7 | 2.6 | 44 |
| Chen2022 | 9.38 | 0.76 | 56 | 10.84 | 1.18 | 53 |
| Wei2016 | 8.67 | 1.35 | 20 | 9.87 | 1.45 | 20 |
| Wang2022 | 6.89 | 1.16 | 60 | 11.9 | 2.35 | 60 |
| Li2016 | 8.67 | 3.65 | 30 | 10.97 | 2.63 | 30 |
| Cai2021 | 8.25 | 1.31 | 43 | 9.89 | 1.24 | 43 |
|  |  |  |  |  |  |  |
|  |  |  |  |  |  |  |
| **TNF-α** |  |  |  |  |  |  |
| study |  |  |  |  |  |  |
|  | mean1 | sd1 | n1 | mean2 | sd2 | n2 |
| Cai2021 | 62.3 | 22.5 | 43 | 88.6 | 29.8 | 43 |
| Tang2020 | 30.42 | 1.63 | 45 | 37.54 | 2.53 | 45 |
|  | 45.56 | 5.11 | 20 | 64.07 | 4.13 | 20 |
