# Supplementary figures and images for: Intraoperative transcutaneous electrical acupoint stimulation combined with anesthesia to prevent postoperative cognitive dysfunction: A systematic review and meta-analysis
Source: PLoS One. 2025 Jan 9;20(1):e0313622. doi: 10.1371/journal.pone.0313622 (PMC11717303; doi:10.1371/journal.pone.0313622)

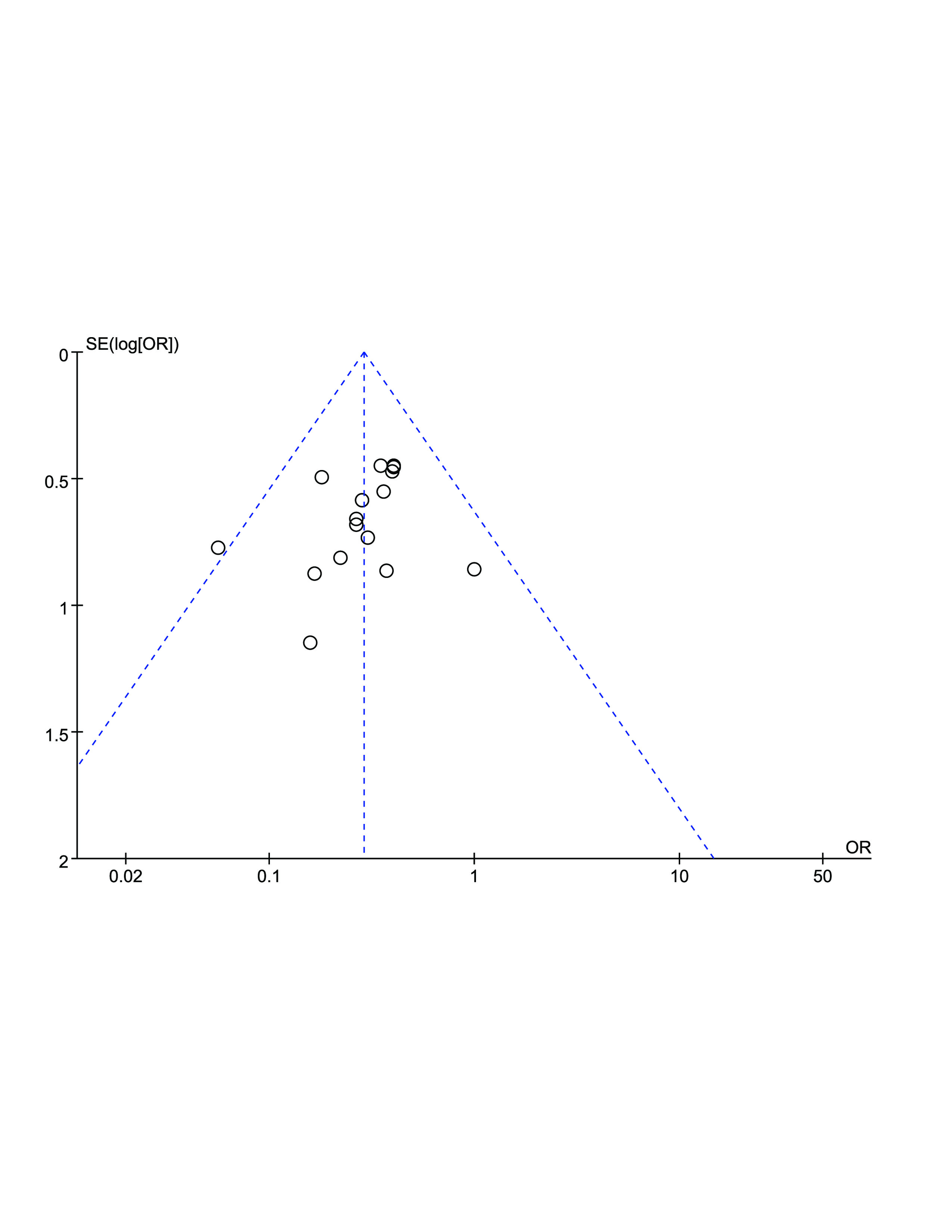

Supplement: S1 Fig — (TIF) [file pone.0313622.s003.tif]

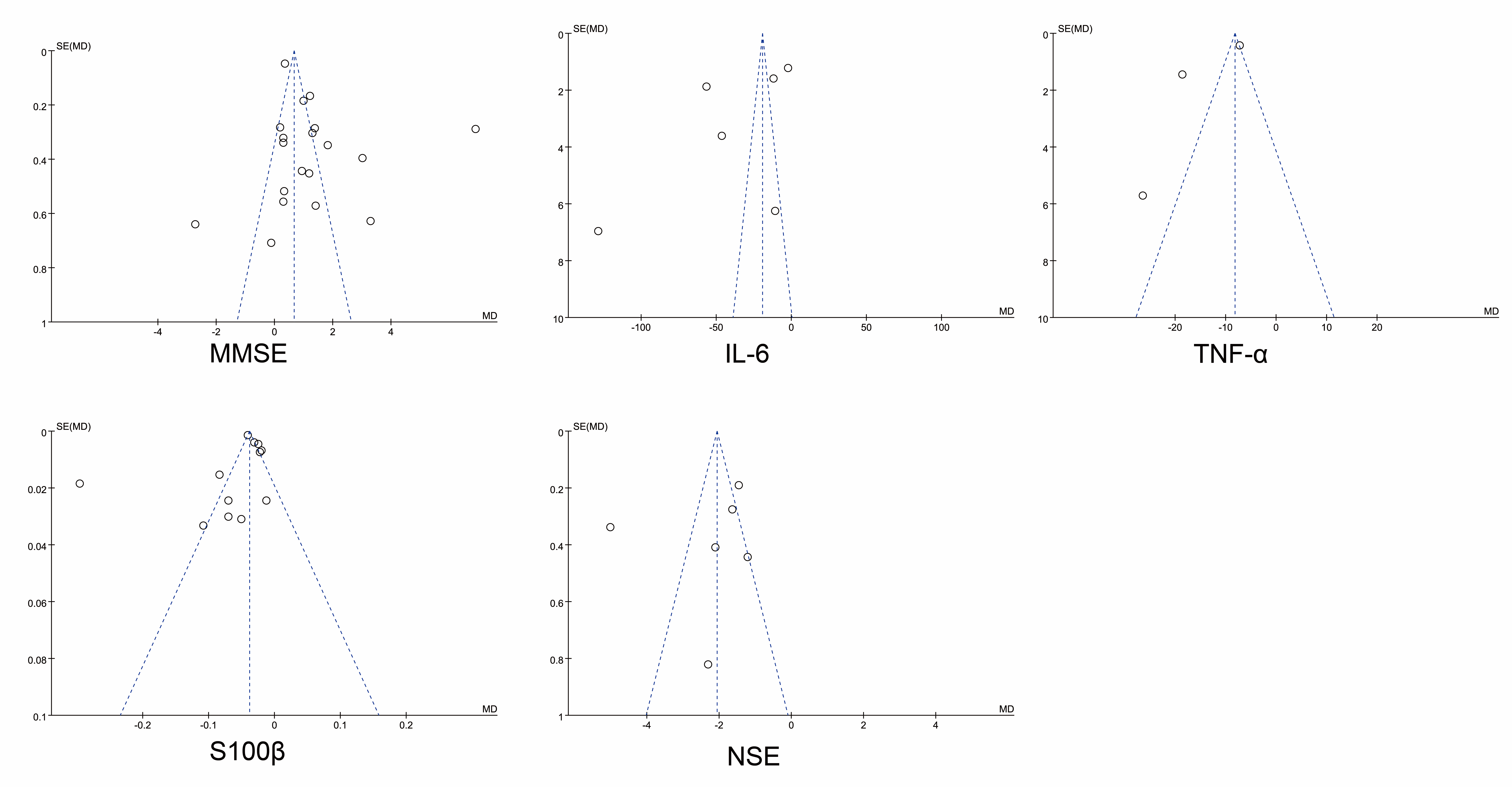

Supplement: S2 Fig — (TIF) [file pone.0313622.s004.tif]
