# Supplementary material for: Intraoperative transcutaneous electrical acupoint stimulation combined with anesthesia to prevent postoperative cognitive dysfunction: A systematic review and meta-analysis
Source: PLoS One. 2025 Jan 9;20(1):e0313622. doi: 10.1371/journal.pone.0313622 (PMC11717303; doi:10.1371/journal.pone.0313622)
Supplement: S1 Table — (DOCX) [file pone.0313622.s005.docx]

**S1 Table. Search strategy**

| **Databases** | **Search terms** | **Date** |
| --- | --- | --- |
| PubMed | (Cognitive Function OR Cognitive Dysfunction OR Cognitive Impairments OR POCD) AND (Transcutaneous Electrical Acupoint Stimulation OR Acupoint Stimulation OR Electric Stimulation)  Search option: All fields | July 20, 2024 |
| Cochrane library | (Cognitive Function OR Cognitive Dysfunction OR Cognitive Impairments OR POCD) AND (Transcutaneous Electrical Acupoint Stimulation OR Acupoint Stimulation OR Electric Stimulation)  Search option: All fields | July 20, 2024 |
| Web of Science | (Cognitive Function OR Cognitive Dysfunction OR Cognitive Impairments OR POCD) AND (Transcutaneous Electrical Acupoint Stimulation OR Acupoint Stimulation OR Electric Stimulation)  Search option: All fields | July 20, 2024 |
| China National Knowledge Infrastructure (CNKI) | (Cognitive Function OR Cognitive Dysfunction OR Cognitive Impairments OR POCD) AND (Transcutaneous Electrical Acupoint Stimulation OR Acupoint Stimulation OR Electric Stimulation)  Search option: All fields | July 20, 2024 |
| Wanfang Data | (Cognitive Function OR Cognitive Dysfunction OR Cognitive Impairments OR POCD) AND (Transcutaneous Electrical Acupoint Stimulation OR Acupoint Stimulation OR Electric Stimulation)  Search option: All fields | July 20, 2024 |
