# Supplementary material for: Intraoperative transcutaneous electrical acupoint stimulation combined with anesthesia to prevent postoperative cognitive dysfunction: A systematic review and meta-analysis
Source: PLoS One. 2025 Jan 9;20(1):e0313622. doi: 10.1371/journal.pone.0313622 (PMC11717303; doi:10.1371/journal.pone.0313622)
Supplement: S2 Table — (DOCX) [file pone.0313622.s006.docx]

S2 Table. All studies identified in the literature search

| NO | Date base | Tital | Year | Atical Type | Reason(s) for Exclusion |
| --- | --- | --- | --- | --- | --- |
| 1 | Pubmed | Hominis Placenta modulates PTSD-like behaviors in SPSS-induced PTSD mice: Regulating energy metabolism and neuronal activity | 2024 | Animal Study | Article type mismatch |
| 2 | Pubmed | Olfactory Three-Needle Electroacupuncture Improved Synaptic Plasticity and Gut Microbiota of SAMP8 Mice by Stimulating Olfactory Nerve | 2024 | Animal Study | Article type mismatch |
| 3 | Pubmed | Effectiveness of Acupressure for Fatigue Alleviation in Patients Receiving Hemodialysis: A Systematic Review and Meta-Analysis of Randomized Controlled Trials | 2024 | Review | Review Exclused |
| 4 | Pubmed | Electroacupuncture Ameliorates Cognitive Impairment by Regulating γ-Amino Butyric Acidergic Interneurons in the Hippocampus of 5 Familial Alzheimer's Disease Mice | 2024 | Animal Study | Missing full text |
| 5 | Pubmed | The research progress of perioperative non-pharmacological interventions on postoperative cognitive dysfunction: a narrative review | 2024 | Review | Review Exclused |
| 6 | Pubmed | Effects of conventional nursing in the operating room combined with transcutaneous electrical acupoint stimulation on postoperative cognitive dysfunction after total knee arthroplasty in elderly patients | 2024 | Clinical trail | Included |
| 7 | Pubmed | Electroacupuncture pretreatment preserves telomerase reverse transcriptase function and alleviates postoperative cognitive dysfunction by suppressing oxidative stress and neuroinflammation in aged mice | 2024 | Animal Study | Missing full text |
| 8 | Pubmed | Electroacupuncture ameliorates cognitive impairment in APP/PS1 mouse by modulating TFEB levels to relieve ALP dysfunction | 2023 | Animal Study | Article type mismatch |
| 9 | Pubmed | Impact of Transcutaneous Electrical Acupoint Stimulation on Delayed Neurocognitive Recovery in Elderly Patients Following Bronchoscopy | 2023 | Clinical trail | Article type mismatch |
| 10 | Pubmed | Needle retaining after electroacupuncture combined with cognitive training for post-stroke cognitive impairment: a multi-center randomized controlled trial | 2023 | Clinical trail | Different intervention methods |
| 11 | Pubmed | Effect of transcutaneous electrical acupoint stimulation on postoperative cognitive function in older patients with lung cancer: A randomized, double-blind, placebo-controlled trial | 2023 | Clinical trail | Included |
| 12 | Pubmed | Efficacy of electroacupuncture stimulating Shenmen (HT7), Baihui (GV20), Sanyinjiao (SP6) on spatial learning and memory deficits in rats with insomnia induced by para-chlorophenylalanine: a single acupoint combined acupoints | 2023 | Animal Study | Article type mismatch |
| 13 | Pubmed | Effects of Electroacupuncture Pretreatment on Long-term Postoperative Cognitive Dysfunction and Hippocampal Inflammation in Aged Rats | 2023 | Animal Study | Missing full text |
| 14 | Pubmed | The neuroprotective and neural circuit mechanisms of acupoint stimulation for cognitive impairment | 2023 | Review | Missing full text |
| 15 | Pubmed | Transcutaneous electrical acupoint stimulation combined with an integrated perioperative nursing program prevents subsyndromal delirium in older patients after joint replacement | 2022 | Clinical trail | Missing full text |
| 16 | Pubmed | Transcutaneous electrical acupoint stimulation for reducing cognitive dysfunction in lumbar spine surgery: A randomized, controlled trail | 2022 | Clinical trail | Missing full text |
| 17 | Pubmed | The efficacy and safety of transcutaneous auricular vagus nerve stimulation in patients with mild cognitive impairment: A double blinded randomized clinical trial. | 2022 | Animal Study | Missing full text |
| 18 | Pubmed | Effect of TEAS combined with general anesthesia on early postoperative cognitive function in elderly patients undergoing single-port thoracoscopic lobectomy | 2022 | Clinical trail | Included |
| 19 | Pubmed | Transcutaneous Electrical Acupoint Stimulation Improves Postoperative Cognitive Function in Senior Patients Undergoing Video-Assisted Thoracoscopic Surgery: A Randomized Controlled Trial | 2022 | Clinical trail | Included |
| 20 | Pubmed | Transcutaneous electrical acupoint stimulation affects older adults' cognition after general anesthesia: A meta-analysis | 2022 | Review | Review Exclused |
| 21 | Pubmed | Electroacupuncture Increases the Hippocampal Synaptic Transmission Efficiency and Long-Term Plasticity to Improve Vascular Cognitive Impairment | 2022 | Animal Study | Missing full text |
| 22 | Pubmed | Electro-Acupuncture Pretreatment Ameliorates Anesthesia and Surgery-Induced Cognitive Dysfunction Via Inhibiting Mitochondrial Injury and nEuroapoptosis in Aged Rats | 2022 | Animal Study | Missing full text |
| 23 | Pubmed | Scalp Stimulation Targets for Neurological Conditions-Evidence from Large-Scale Meta-Analyses | 2022 | Review | Review Exclused |
| 24 | Pubmed | Perioperative Acupuncture Optimizes Surgical Outcomes: Theory, Clinical Practice and Future Perspectives | 2022 | Review | Review Exclused |
| 25 | Pubmed | Transcutaneous electrical acupoint stimulation for postoperative cognitive dysfunction in geriatric patients with gastrointestinal tumor: a randomized controlled trial | 2021 | Clinical trail | Different intervention methods |
| 26 | Pubmed | Factors contributing to cognitive improvement effects of acupuncture in patients with mild cognitive impairment: a pilot randomized controlled trial | 2021 | Clinical trail | Different intervention methods |
| 27 | Pubmed | Effects of Transcutaneous Electrical Acupoint Stimulation on Postoperative Cognitive Decline in Elderly Patients: A Pilot Study | 2021 | Clinical trail | Included |
| 28 | Pubmed | Application of transcutaneous electrical acupoint stimulation in perioperative period | 2021 | Review | Review Exclused |
| 29 | Pubmed | Effect of magnetic stimulation of Shenmen point on cognitive function of chronic insomnia: A randomized controlled clinical trial | 2020 | Clinical trail | Different intervention methods |
| 30 | Pubmed | Assessor- and participant-blinded, randomized controlled trial of dense cranial electroacupuncture stimulation plus body acupuncture for neuropsychiatric sequelae of stroke | 2020 | Clinical trail | Missing full text |
| 31 | Pubmed | Effect of electroacupuncture on cognitive functions, hippocampal HIF-1α and neurons apoptosis in postoperative cognitive dysfunction rats | 2020 | Animal Study | Article type mismatch |
| 32 | Pubmed | Effects of electroacupuncture on postoperative cognitive dysfunction and AngⅡ/AT1R in the hippocampus in aging rats induced by D-galactose | 2017 | Review | Review Exclused |
| 33 | Pubmed | Individualized scalp acupuncture for motor dysfunction in stroke: a randomized controlled trial | 2017 | Clinical trail | Missing full text |
| 34 | Pubmed | Effect of Dexmedetomidine Combined Electrical Stimulation on Coanitive Function of Patients Receiving Extracerebral Intervention | 2016 | Clinical trail | Different intervention methods |
| 35 | Pubmed | TEAS for prevention and treatment of orthodontic toothache and oral dysfunction: a randomized controlled trial | 2016 | Clinical trail | Missing full text |
| 36 | Pubmed | Electroacupuncture for older adults with mild cognitive impairment: study protocol for a randomized controlled trial | 2015 | Clinical trail | Missing full text |
| 37 | Pubmed | Acupuncture stimulation improves scopolamine-induced cognitive impairment via activation of cholinergic system and regulation of BDNF and CREB expressions in rats | 2014 | Animal Study | Article type mismatch |
| 38 | Pubmed | Impacts of the different frequencies of electroacupunctrue on cognitive function in patients after abdominal operation under compound anesthesia of acupuncture and drugs | 2013 | Clinical trail | Included |
| 39 | embase | Olfactory Three-Needle Electroacupuncture Improved Synaptic Plasticity and Gut Microbiota of SAMP8 Mice by Stimulating Olfactory Nerve | 2024 | Animal Study | Duplication excluded |
| 40 | embase | Effectiveness of Acupressure for Fatigue Alleviation in Patients Receiving Hemodialysis: A Systematic Review and Meta-Analysis of Randomized Controlled Trials | 2024 | Review | Duplication excluded |
| 41 | embase | Electroacupuncture Ameliorates Cognitive Impairment by Regulating γ-Amino Butyric Acidergic Interneurons in the Hippocampus of 5 Familial Alzheimer's Disease Mice | 2024 | Animal Study | Duplication excluded |
| 42 | embase | The research progress of perioperative non-pharmacological interventions on postoperative cognitive dysfunction: a narrative review | 2024 | Review | Duplication excluded |
| 43 | embase | Effects of conventional nursing in the operating room combined with transcutaneous electrical acupoint stimulation on postoperative cognitive dysfunction after total knee arthroplasty in elderly patients | 2024 | Clinical trail | Duplication excluded |
| 44 | embase | Electroacupuncture ameliorates cognitive impairment in APP/PS1 mouse by modulating TFEB levels to relieve ALP dysfunction | 2023 | Animal Study | Duplication excluded |
| 45 | embase | Impact of Transcutaneous Electrical Acupoint Stimulation on Delayed Neurocognitive Recovery in Elderly Patients Following Bronchoscopy | 2023 | Clinical trail | Duplication excluded |
| 46 | embase | Efficacy of electroacupuncture stimulating Shenmen (HT7), Baihui (GV20), Sanyinjiao (SP6) on spatial learning and memory deficits in rats with insomnia induced by para-chlorophenylalanine: a single acupoint combined acupoints | 2023 | Animal Study | Duplication excluded |
| 47 | embase | The neuroprotective and neural circuit mechanisms of acupoint stimulation for cognitive impairment | 2023 | Review | Duplication excluded |
| 48 | embase | Transcutaneous electrical acupoint stimulation combined with an integrated perioperative nursing program prevents subsyndromal delirium in older patients after joint replacement | 2022 | Clinical trail | Duplication excluded |
| 49 | embase | Transcutaneous electrical acupoint stimulation for reducing cognitive dysfunction in lumbar spine surgery: A randomized, controlled trail | 2022 | Clinical trail | Duplication excluded |
| 50 | embase | The efficacy and safety of transcutaneous auricular vagus nerve stimulation in patients with mild cognitive impairment: A double blinded randomized clinical trial. | 2022 | Animal Study | Duplication excluded |
| 51 | embase | Effect of TEAS combined with general anesthesia on early postoperative cognitive function in elderly patients undergoing single-port thoracoscopic lobectomy | 2022 | Clinical trail | Duplication excluded |
| 52 | embase | Perioperative Acupuncture Optimizes Surgical Outcomes: Theory, Clinical Practice and Future Perspectives | 2022 | Review | Duplication excluded |
| 53 | embase | Transcutaneous electrical acupoint stimulation for postoperative cognitive dysfunction in geriatric patients with gastrointestinal tumor: a randomized controlled trial | 2021 | Clinical trail | Duplication excluded |
| 54 | embase | Factors contributing to cognitive improvement effects of acupuncture in patients with mild cognitive impairment: a pilot randomized controlled trial | 2021 | Clinical trail | Duplication excluded |
| 55 | embase | Electroacupuncture for older adults with mild cognitive impairment: study protocol for a randomized controlled trial | 2015 | Clinical trail | Duplication excluded |
| 56 | embase | Acupuncture stimulation improves scopolamine-induced cognitive impairment via activation of cholinergic system and regulation of BDNF and CREB expressions in rats | 2014 | Animal Study | Duplication excluded |
| 57 | Cochrane Library | Hominis Placenta modulates PTSD-like behaviors in SPSS-induced PTSD mice: Regulating energy metabolism and neuronal activity | 2024 | Animal Study | Duplication excluded |
| 58 | Cochrane Library | Olfactory Three-Needle Electroacupuncture Improved Synaptic Plasticity and Gut Microbiota of SAMP8 Mice by Stimulating Olfactory Nerve | 2024 | Animal Study | Duplication excluded |
| 59 | Cochrane Library | Effectiveness of Acupressure for Fatigue Alleviation in Patients Receiving Hemodialysis: A Systematic Review and Meta-Analysis of Randomized Controlled Trials | 2024 | Review | Duplication excluded |
| 60 | Cochrane Library | Electroacupuncture Ameliorates Cognitive Impairment by Regulating γ-Amino Butyric Acidergic Interneurons in the Hippocampus of 5 Familial Alzheimer's Disease Mice | 2024 | Animal Study | Duplication excluded |
| 61 | Cochrane Library | The research progress of perioperative non-pharmacological interventions on postoperative cognitive dysfunction: a narrative review | 2024 | Review | Duplication excluded |
| 62 | Cochrane Library | Effects of conventional nursing in the operating room combined with transcutaneous electrical acupoint stimulation on postoperative cognitive dysfunction after total knee arthroplasty in elderly patients | 2024 | Clinical trail | Duplication excluded |
| 63 | Cochrane Library | Electroacupuncture ameliorates cognitive impairment in APP/PS1 mouse by modulating TFEB levels to relieve ALP dysfunction | 2023 | Animal Study | Duplication excluded |
| 64 | Cochrane Library | Impact of Transcutaneous Electrical Acupoint Stimulation on Delayed Neurocognitive Recovery in Elderly Patients Following Bronchoscopy | 2023 | Clinical trail | Duplication excluded |
| 65 | Cochrane Library | Efficacy of electroacupuncture stimulating Shenmen (HT7), Baihui (GV20), Sanyinjiao (SP6) on spatial learning and memory deficits in rats with insomnia induced by para-chlorophenylalanine: a single acupoint combined acupoints | 2023 | Animal Study | Duplication excluded |
| 66 | Cochrane Library | The neuroprotective and neural circuit mechanisms of acupoint stimulation for cognitive impairment | 2023 | Review | Duplication excluded |
| 67 | Cochrane Library | Transcutaneous electrical acupoint stimulation combined with an integrated perioperative nursing program prevents subsyndromal delirium in older patients after joint replacement | 2022 | Clinical trail | Duplication excluded |
| 68 | Cochrane Library | Transcutaneous electrical acupoint stimulation for reducing cognitive dysfunction in lumbar spine surgery: A randomized, controlled trail | 2022 | Clinical trail | Duplication excluded |
| 69 | Cochrane Library | The efficacy and safety of transcutaneous auricular vagus nerve stimulation in patients with mild cognitive impairment: A double blinded randomized clinical trial. | 2022 | Animal Study | Duplication excluded |
| 70 | Cochrane Library | Effect of TEAS combined with general anesthesia on early postoperative cognitive function in elderly patients undergoing single-port thoracoscopic lobectomy | 2022 | Clinical trail | Duplication excluded |
| 71 | Cochrane Library | Perioperative Acupuncture Optimizes Surgical Outcomes: Theory, Clinical Practice and Future Perspectives | 2022 | Review | Duplication excluded |
| 72 | Cochrane Library | Transcutaneous electrical acupoint stimulation for postoperative cognitive dysfunction in geriatric patients with gastrointestinal tumor: a randomized controlled trial | 2021 | Clinical trail | Duplication excluded |
| 73 | Cochrane Library | Effect of magnetic stimulation of Shenmen point on cognitive function of chronic insomnia: A randomized controlled clinical trial | 2020 | Clinical trail | Duplication excluded |
| 74 | Cochrane Library | Electroacupuncture for older adults with mild cognitive impairment: study protocol for a randomized controlled trial | 2015 | Clinical trail | Duplication excluded |
| 75 | Cochrane Library | Acupuncture stimulation improves scopolamine-induced cognitive impairment via activation of cholinergic system and regulation of BDNF and CREB expressions in rats | 2014 | Animal Study | Duplication excluded |
| 76 | Web of Science | Electroacupuncture pretreatment preserves telomerase reverse transcriptase function and alleviates postoperative cognitive dysfunction by suppressing oxidative stress and neuroinflammation in aged mice | 2024 | Animal Study | Duplication excluded |
| 77 | Web of Science | Electroacupuncture ameliorates cognitive impairment in APP/PS1 mouse by modulating TFEB levels to relieve ALP dysfunction | 2023 | Animal Study | Duplication excluded |
| 78 | Web of Science | Impact of Transcutaneous Electrical Acupoint Stimulation on Delayed Neurocognitive Recovery in Elderly Patients Following Bronchoscopy | 2023 | Clinical trail | Duplication excluded |
| 79 | Web of Science | Needle retaining after electroacupuncture combined with cognitive training for post-stroke cognitive impairment: a multi-center randomized controlled trial | 2023 | Clinical trail | Duplication excluded |
| 80 | Web of Science | Effect of transcutaneous electrical acupoint stimulation on postoperative cognitive function in older patients with lung cancer: A randomized, double-blind, placebo-controlled trial | 2023 | Clinical trail | Duplication excluded |
| 81 | Web of Science | Transcutaneous electrical acupoint stimulation combined with an integrated perioperative nursing program prevents subsyndromal delirium in older patients after joint replacement | 2022 | Clinical trail | Duplication excluded |
| 82 | Web of Science | Transcutaneous electrical acupoint stimulation for reducing cognitive dysfunction in lumbar spine surgery: A randomized, controlled trail | 2022 | Clinical trail | Duplication excluded |
| 83 | Web of Science | The efficacy and safety of transcutaneous auricular vagus nerve stimulation in patients with mild cognitive impairment: A double blinded randomized clinical trial. | 2022 | Animal Study | Duplication excluded |
| 84 | Web of Science | Factors contributing to cognitive improvement effects of acupuncture in patients with mild cognitive impairment: a pilot randomized controlled trial | 2021 | Clinical trail | Duplication excluded |
| 85 | Web of Science | Effect of magnetic stimulation of Shenmen point on cognitive function of chronic insomnia: A randomized controlled clinical trial | 2020 | Clinical trail | Duplication excluded |
| 86 | Web of Science | Assessor- and participant-blinded, randomized controlled trial of dense cranial electroacupuncture stimulation plus body acupuncture for neuropsychiatric sequelae of stroke | 2020 | Clinical trail | Duplication excluded |
| 87 | Web of Science | Electroacupuncture for older adults with mild cognitive impairment: study protocol for a randomized controlled trial | 2015 | Clinical trail | Duplication excluded |
| 88 | CNKI | Study on the effect of percutaneous acupoint electrical stimulation during perioperative period to prevent cognitive impairment after total knee arthroplasty in the elderly | 2024 | Clinical trail | Different intervention methods |
| 89 | CNKI | Effect of electro-acupuncture intervention on mitochondrial fission in diabetic mice with postoperative cognitive dysfunction | 2023 | Animal Study | Article type mismatch |
| 90 | CNKI | Effects of Electroacupuncture Pretreatment on Long-term Postoperative Cognitive Dysfunction and Hippocampal Inflammation in Aged Rats | 2023 | Animal Study | Duplication excluded |
| 91 | CNKI | Effect of Electroacupuncture on Inflammation Response and Ferroptosis in Rats Modeling Postoperative Cognitive Dysfunction | 2023 | Animal Study | Article type mismatch |
| 92 | CNKI | Influence of Transcutaneous Electrical Acupoint Stimulation on Cognitive Function after Hip Replacement in the Elderly:a Meta-analysis | 2023 | Review | Review Exclused |
| 93 | CNKI | Effects of electroacupuncture on postoperative cognitive dysfunction and peripheral inflammatory factors in elderly patients after gastrointestinal surgery | 2023 | Clinical trail | Different intervention methods |
| 94 | CNKI | Application effect of acupointelectrical stimulation combined with general anesthesia in elderly patients undergoing laparoscopic radical resection of colorectal cancer | 2023 | Clinical trail | Different intervention methods |
| 95 | CNKI | Impact of electroacupuncture pretreatment on the cognitive function in older patients after holmium laser lithotripsy under flexible ureteroscope | 2023 | Clinical trail | Different intervention methods |
| 96 | CNKI | Improvement Effect of Acupuncture on Postoperative Cognitive Dysfunction in Aged Mice and Its Effects on ApoE and PSD－95 | 2022 | Review | Review Exclused |
| 97 | CNKI | Research progress in application of transcutaneous electrical acupoint stimulation in anesthesia | 2022 | Review | Review Exclused |
| 98 | CNKI | Transcutaneous Electrical Acupoint Stimulation Improves Postoperative Cognitive Function in Senior Patients Undergoing Video-Assisted Thoracoscopic Surgery: A Randomized Controlled Trial | 2022 | Clinical trail | Duplication excluded |
| 99 | CNKI | Effect of Electroacupuncture At Yongquan, Shenmen and Baihui Points on Postoperative Cognitive Dysfunction in Elderly Patients | 2022 | Clinical trail | Different intervention methods |
| 100 | CNKI | Effects of Electroacupuncture at Hegu (LI4) and Neiguan (PC6) Combined with Intravenous General Anesthesia on Cerebral Oxygen Metabolism and Cognitive Function in Patients Undergoing Cervical Lymph NodeLesion Clearance Surgery | 2022 | Clinical trail | Different intervention methods |
| 101 | CNKI | Effect of Transcutaneous Electrical Acupoint Stimulation on Postoperative Cognitive Function in Elderly Patients Undergoing Hip Fracture Surgery | 2022 | Clinical trail | Included |
| 102 | CNKI | The Effect and Mechanism of Electroacupuncture on Postoperative Cognitive Dysfunction of Rat Based on HIF-1α/VEGF Axis | 2021 | Animal Study | Article type mismatch |
| 103 | CNKI | Application of transcutaneous electrical acupoint stimulation in perioperative period | 2021 | Review | Duplication excluded |
| 104 | CNKI | Effects of Percutaneous Acupoint Electrical Stimulation on Postoperative Cognitive Dysfunction in Older Patients Undergoing Hip Replacement | 2021 | Clinical trail | Included |
| 105 | CNKI | The effect of transcutaneous acupoint electrical stimulation combined with dexmedetomidine on the cognitive function of elderly patients after hip surgery | 2021 | Clinical trail | Different intervention methods |
| 106 | CNKI | Effects of preoperative TEAS prewarming strategy on postanesthesia recovery and cognitive function of elderly patients undergoing video-assisted thoracoscopic lobectomy | 2021 | Clinical trail | Missing full text |
| 107 | CNKI | Effect of Perioperative Transcutaneous Electrical Acupoint Stimulation on the Early Postoperative Rehabilitation for Elderly Patients with Gastrointestinal Tumor | 2021 | Clinical trail | Different intervention methods |
| 108 | CNKI | Effects of Transcutaneous Electrical Acupoint Stimulation on Postoperative Cognitive Decline in Elderly Patients: A Pilot Study | 2021 | Clinical trail | Duplication excluded |
| 109 | CNKI | Study on the Effect of Electroacupuncture Combined with Dexmedetomidine on Intraoperative Stress in Elderly Orthopedic Patients | 2021 | Clinical trail | Different intervention methods |
| 110 | CNKI | Effect of TEAS combined with cognitive behavior training on POCD of hip fracture | 2021 | Clinical trail | Missing full text |
| 111 | CNKI | Effects of Transcutaneous Acupoint Electrical Stimulation Pretreatment on Postoperative Cognitive Dysfunction and Inflammatory Factors in Elderly Patients | 2021 | Clinical trail | Different intervention methods |
| 112 | CNKI | Effect of electroacupuncture on cognitive functions, hippocampal HIF-1α and neurons apoptosis in postoperative cognitive dysfunction rats | 2020 | Animal Study | Duplication excluded |
| 113 | CNKI | Effect of Electroacupuncture on the Cognitive Function and Expression of α7nAChR Receptor in the Hippocampus of Rats with Postoperative Cognitive Dysfunction | 2020 | Animal Study | Article type mismatch |
| 114 | CNKI | Effects of Transcutaneous Acupoint Electrical Stimulation Combined with General Anesthesia on Inflammatory Factors, T Cell Subsets and Cognitive Function in Patients Undergoing Laparoscopic Radical Resection of Colorectal Cancer | 2020 | Clinical trail | Included |
| 115 | CNKI | Effect of electroacupuncture on cognitive functions, hippocampal HIF-1α and neurons apoptosis in postoperative cognitive dysfunction rats | 2020 | Animal Study | Missing full text |
| 116 | CNKI | Effect of Electroacupuncture on Cognitive Function and Hippocampal Glial Cells in Partially Hepatectomized Rats | 2020 | Animal Study | Missing full text |
| 117 | CNKI | Effect of Electroacupuncture at "Siguan" Points on Postoperative Cognitive Function and Serum HIF-1Α in Elderly Patients with Hip Replacement | 2019 | Clinical trail | Different intervention methods |
| 118 | CNKI | Electroacupuncture improves postoperative cognitive dysfunction by enhancing autophagy in aged rats | 2019 | Animal Study | Missing full text |
| 119 | CNKI | Effect of Transcutaneous Electrical Acupoint Stimulation on Cerebral Oxygen Metabolism, Postoperative Cognitive Function and Analgesic Effect in Elderly Patients Undergoing Hip Replacement | 2019 | Clinical trail | Included |
| 120 | CNKI | Effect of transcutaneous electrical acupoint stimulation on postoperative cognitive dysfunction in elderly patients | 2019 | Clinical trail | Missing full text |
| 121 | CNKI | Research Progress of Anesthesia-related Measures to Prevent Postoperative Cognitive Dysfunction in Elderly Pa tients Undergoing Hip Joint Surgery | 2019 | Review | Review Exclused |
| 122 | CNKI | Effect of transcutaneous electrical acupuncture stimulation on postoperative cognitive dysfunction in elderly patients undergoing controlled hypotension | 2019 | Clinical trail | Missing full text |
| 123 | CNKI | The pathogenesis of postoperative cognitive dysfunction in elderly patients | 2019 | Review | Review Exclused |
| 124 | CNKI | Effects of transcutaneous acupoint electrical stimulation assisted with general anesthesia on postoperative immune function and cognitive function in elderly patients undergoing cardiac surgery | 2019 | Clinical trail | Included |
| 125 | CNKI | Effects of Acupuncture Anesthesia and Gas Anesthesia on Elderly Patients with Postoperative Cognitive Dysfunction and NSE, S100β Protein Levels Chang | 2018 | Clinical trail | Different outcome indicators |
| 126 | CNKI | Effects of early transcutaneous acupoint electrical stimulation on postoperative cognition of elderly patients with hip fractures | 2018 | Clinical trail | Missing full text |
| 127 | CNKI | Effect of Electroacupuncture Stimulation on Postoperative Cognitive Disorder of Elderly Patients after General Anesthesia | 2018 | Clinical trail | Different intervention methods |
| 128 | CNKI | Research Progress of Traditional Chinese Medicine and Postoperative Cognitive Dysfunction in Elderly Patients | 2018 | Review | Review Exclused |
| 129 | CNKI | Effect of Transcutaneous Electrical Acupoint Stimulation Combined with Dexamethasone on Postoperative Cognitive function in Elderly Patients with Benign Prostatic Hyperplasia | 2018 | Clinical trail | Different intervention methods |
| 130 | CNKI | Effects of transcutaneous electrical acupoint stimulation on quality of recovery during early period after laparoscopic cholecystectomy | 2018 | Clinical trail | Included |
| 131 | CNKI | Effect of electroacupuncture at “four close” points on cognitive function in elderly patients with hip replacement | 2017 | Clinical trail | Different intervention methods |
| 132 | CNKI | Effect of percutaneous acupoint electrical stimulation on postoperative cognitive function in elderly patients with colorectal cancer | 2017 | Clinical trail | Missing full text |
| 133 | CNKI | Effect and mechanism of electroacupuncture preconditioning on postoperative cognitive function in aged rats | 2017 | Animal Study | Missing full text |
| 134 | CNKI | Effect of Electroacupuncture on Cognition and Hippocampal TNF-α and IL-1β Expressions in Rats with Postoperative Cognitive Dysfunction | 2017 | Review | Review Exclused |
| 135 | CNKI | Effects of electroacupuncture on postoperative cognitive dysfunction and AngⅡ/AT1R in the hippocampus in aging rats induced by D-galactose | 2017 | Animal Study | Duplication excluded |
| 136 | CNKI | Effect of TEAS on Cognitive Function and Serum S100 Beta Protein in Senile Patients after Cholecystectomy | 2017 | Clinical trail | Missing full text |
| 137 | CNKI | Effect of Electroacupuncture on Early Postoperative Cognitive Dysfunction in Elderly Patients Undergoing General Anesthesia | 2017 | Clinical trail | Missing full text |
| 138 | CNKI | Research on the Effect of Electro-acupuncture Therapy of Traditional Chinese Medicine on the Recovery of Cognitive Function in Elderly Patients after General Anesthesia | 2017 | Clinical trail | Missing full text |
| 139 | CNKI | Effect of Clinical Nursing of TCM on the Recovery of Cognitive Function after Operation under General Anesthesia in the Elderly | 2017 | Clinical trail | Different intervention methods |
| 140 | CNKI | Effects of electrotherapy on postoperative cognitive dysfunction in elderly patients with insulin-resistant joint replacement | 2017 | Clinical trail | Different intervention methods |
| 141 | CNKI | Individualized scalp acupuncture for motor dysfunction in stroke: a randomized controlled trial | 2017 | Clinical trail | Duplication excluded |
| 142 | CNKI | Effect of Different Frequency Transcutaneous Electrical Acupoint Stimulation Treated on the Postoperative Cognitive Function of Patients undergoing Gynecological Laparoscopy | 2017 | Clinical trail | Different intervention methods |
| 143 | CNKI | Effect of Combined Acupuncture and Drug Anesthesia Combined With Electrical Stimulation on Cognitive Function and Serum S-100β Protein in Patients with Abdominal Surgery | 2017 | Clinical trail | Different intervention methods |
| 144 | CNKI | Effects of transcutaneous electrical acupoint stimulation on early postoperative cognitive function in patients with transsphenoidal surgery | 2017 | Clinical trail | Different intervention methods |
| 145 | CNKI | Effect of Transcutaneous Electrical Stimulation at Acupoints on Postoperative Cognitive Function of Gynecological Laparoscopy Patients | 2016 | Clinical trail | Included |
| 146 | CNKI | Effect of Dexmedetomidine Combined Electrical Stimulation on Coanitive Function of Patients Receiving Extracerebral Intervention | 2016 | Clinical trail | Duplication excluded |
| 147 | CNKI | TEAS for prevention and treatment of orthodontic toothache and oral dysfunction: a randomized controlled trial | 2016 | Clinical trail | Duplication excluded |
| 148 | CNKI | Effect of Mental Tri-needle Electro-acupuncture on Postoperative Cognitive Function in Elderly Patients After Hip Joint Replacement Surgery | 2016 | Clinical trail | Different intervention methods |
| 149 | CNKI | Effects of electroacupuncture combined with epidural anesthesia on cognitive function in elderly patients after abdominal surgery | 2016 | Clinical trail | Missing full text |
| 150 | CNKI | Clinical observation of cognitive dysfunction in elderly patients after abdominal operation under general anesthesia treated with percutaneous acupoint electrical stimulation | 2016 | Clinical trail | Different intervention methods |
| 151 | CNKI | Effect of Transcutaneous Electrical Acupoint Stimulation on Postoperative Cognitive Function in Patients Undergoing Radical Thoracoscopic Lung Cancer Operation | 2016 | Clinical trail | Included |
| 152 | CNKI | Effects of Transcutaneous Acupoint Electrical Stimulation and Dexmedetomidine on Postoperative Cognitive Dysfunction in Patients Underwent Laparoscopic Surgery | 2016 | Clinical trail | Included |
| 153 | CNKI | Effect of percutaneous acupoint electrical stimulation on postoperative cognitive function in elderly patients with colorectal cancer | 2016 | Clinical trail | Included |
| 154 | CNKI | Effects of Methylprednisolone Combined with Transcutaneous AcupiontElectric Stimulation on Postoperative Cognitive Dysfunction in Elderly Patients | 2016 | Clinical trail | Included |
| 155 | CNKI | Effect and Mechanism of Acupuncture on Postoperative Cognitive Dysfunction in Aged Rats | 2015 | Animal Study | Article type mismatch |
| 156 | CNKI | Effect of TEAS Combined with General Anesthesia on Cognitive Function of Patients Undergoing Gynaecological Laparoscopy | 2015 | Clinical trail | Included |
| 157 | CNKI | Impacts of electrical acupoint stimulation on postoperative cognitive dysfunction and inflammation cytokines in elderly patients | 2015 | Clinical trail | Different intervention methods |
| 158 | CNKI | Effect of Transcutaneous Acupoint Electrical Stimulation on Postoperative Cognitive Function in Elderly Patients Undergoing Laparoscopic Resection of Rectal Cancer | 2015 | Clinical trail | Included |
| 159 | CNKI | Effects of electroacupuncture preconditioning on postoperative cognitive dysfunction in elderly patients undergoing joint replacement | 2014 | Clinical trail | Missing full text |
| 160 | CNKI | Effect of Electroacupuncture on Middle-aged and Elderly POCD Rats’HPA Axis and Cognitive Function | 2014 | Review | Review Exclused |
| 161 | CNKI | Effects of transcutaneous electrical acupoint stimulation combined with inhalation anesthesia on postoperative cognitive function of aged patients with laparoscopic cholecystectomy | 2014 | Clinical trail | Missing full text |
| 162 | CNKI | Clinical Observation of Electroacupuncture on Postoperative  Cognitive Dysfunction in Elderly Patients | 2014 | Clinical trail | Missing full text |
| 163 | CNKI | Impacts of the different frequencies of electroacupunctrue on cognitive function in patients after abdominal operation under compound anesthesia of acupuncture and drugs | 2013 | Clinical trail | Duplication excluded |
| 164 | CNKI | Influences of acupuncture anesthesia on postoperative cognitive dysfunction and S-100βprotein level of the elderly patients of colorectal cancer resection | 2013 | Clinical trail | Different intervention methods |
| 165 | CNKI | Effects of Electroacupuncture Assistant General Anesthesia on Postoperative Cognitive Dysfunction of Aged Patients | 2012 | Clinical trail | Missing full text |
| 166 | CNKI | Effects of Electroacupuncture on Neiguan on postoperative cognitive dysfunction in patients undergoing Off-pump coronary artery bypass grafting | 2011 | Clinical trail | Missing full text |
| 167 | CNKI | Effect of Controlled Hypotension Combined with Transcutaneous Electrical Acupoint Stimulation on Early Postoperative Cognitive Dysfunction in Spinal Surgery | 2011 | Clinical trail | Missing full text |
| 168 | CNKI | Effect of Transcutaneous Acupoint Electrical Stimulation on Lipid Peroxidation and Cognitive Function in Patients Experiencing Craniotomy | 2009 | Clinical trail | Included |
| 169 | WanFangdate | Effect of electro-acupuncture intervention on mitochondrial fission in diabetic mice with postoperative cognitive dysfunction | 2023 | Animal Study | Duplication excluded |
| 170 | WanFangdate | Effects of Electroacupuncture Pretreatment on Long-term Postoperative Cognitive Dysfunction and Hippocampal Inflammation in Aged Rats | 2023 | Animal Study | Duplication excluded |
| 171 | WanFangdate | Effect of Electroacupuncture on Inflammation Response and Ferroptosis in Rats Modeling Postoperative Cognitive Dysfunction | 2023 | Animal Study | Duplication excluded |
| 172 | WanFangdate | Application effect of acupointelectrical stimulation combined with general anesthesia in elderly patients undergoing laparoscopic radical resection of colorectal cancer | 2023 | Clinical trail | Duplication excluded |
| 173 | WanFangdate | Impact of electroacupuncture pretreatment on the cognitive function in older patients after holmium laser lithotripsy under flexible ureteroscope | 2023 | Clinical trail | Duplication excluded |
| 174 | WanFangdate | Improvement Effect of Acupuncture on Postoperative Cognitive Dysfunction in Aged Mice and Its Effects on ApoE and PSD－95 | 2022 | Animal Study | Duplication excluded |
| 175 | WanFangdate | Research progress in application of transcutaneous electrical acupoint stimulation in anesthesia | 2022 | Review | Duplication excluded |
| 176 | WanFangdate | Transcutaneous Electrical Acupoint Stimulation Improves Postoperative Cognitive Function in Senior Patients Undergoing Video-Assisted Thoracoscopic Surgery: A Randomized Controlled Trial | 2022 | Clinical trail | Duplication excluded |
| 177 | WanFangdate | Effect of Electroacupuncture At Yongquan, Shenmen and Baihui Points on Postoperative Cognitive Dysfunction in Elderly Patients | 2022 | Clinical trail | Duplication excluded |
| 178 | WanFangdate | Effects of Electroacupuncture at Hegu (LI4) and Neiguan (PC6) Combined with Intravenous General Anesthesia on Cerebral Oxygen Metabolism and Cognitive Function in Patients Undergoing Cervical Lymph NodeLesion Clearance Surgery | 2022 | Clinical trail | Duplication excluded |
| 179 | WanFangdate | Application of transcutaneous electrical acupoint stimulation in perioperative period | 2021 | Review | Duplication excluded |
| 180 | WanFangdate | Effects of Percutaneous Acupoint Electrical Stimulation on Postoperative Cognitive Dysfunction in Older Patients Undergoing Hip Replacement | 2021 | Clinical trail | Duplication excluded |
| 181 | WanFangdate | The effect of transcutaneous acupoint electrical stimulation combined with dexmedetomidine on the cognitive function of elderly patients after hip surgery | 2021 | Clinical trail | Duplication excluded |
| 182 | WanFangdate | Effects of preoperative TEAS prewarming strategy on postanesthesia recovery and cognitive function of elderly patients undergoing video-assisted thoracoscopic lobectomy | 2021 | Clinical trail | Duplication excluded |
| 183 | WanFangdate | Effect of Perioperative Transcutaneous Electrical Acupoint Stimulation on the Early Postoperative Rehabilitation for Elderly Patients with Gastrointestinal Tumor | 2021 | Clinical trail | Duplication excluded |
| 184 | WanFangdate | Effects of Transcutaneous Electrical Acupoint Stimulation on Postoperative Cognitive Decline in Elderly Patients: A Pilot Study | 2021 | Clinical trail | Duplication excluded |
| 185 | WanFangdate | Study on the Effect of Electroacupuncture Combined with Dexmedetomidine on Intraoperative Stress in Elderly Orthopedic Patients | 2021 | Clinical trail | Duplication excluded |
| 186 | WanFangdate | Effect of electroacupuncture on cognitive functions, hippocampal HIF-1α and neurons apoptosis in postoperative cognitive dysfunction rats | 2020 | Animal Study | Duplication excluded |
| 187 | WanFangdate | Effect of Electroacupuncture on the Cognitive Function and Expression of α7nAChR Receptor in the Hippocampus of Rats with Postoperative Cognitive Dysfunction | 2020 | Animal Study | Duplication excluded |
| 188 | WanFangdate | Effects of Transcutaneous Acupoint Electrical Stimulation Combined with General Anesthesia on Inflammatory Factors, T Cell Subsets and Cognitive Function in Patients Undergoing Laparoscopic Radical Resection of Colorectal Cancer | 2020 | Clinical trail | Duplication excluded |
| 189 | WanFangdate | Effect of Electroacupuncture at "Siguan" Points on Postoperative Cognitive Function and Serum HIF-1Α in Elderly Patients with Hip Replacement | 2019 | Clinical trail | Duplication excluded |
| 190 | WanFangdate | Electroacupuncture improves postoperative cognitive dysfunction by enhancing autophagy in aged rats | 2019 | Animal Study | Duplication excluded |
| 191 | WanFangdate | Effect of transcutaneous electrical acupoint stimulation on postoperative cognitive dysfunction in elderly patients | 2019 | Clinical trail | Duplication excluded |
| 192 | WanFangdate | Research Progress of Anesthesia-related Measures to Prevent Postoperative Cognitive Dysfunction in Elderly Pa tients Undergoing Hip Joint Surgery | 2019 | Review | Duplication excluded |
| 193 | WanFangdate | Effect of transcutaneous electrical acupuncture stimulation on postoperative cognitive dysfunction in elderly patients undergoing controlled hypotension | 2019 | Clinical trail | Duplication excluded |
| 194 | WanFangdate | Effects of Acupuncture Anesthesia and Gas Anesthesia on Elderly Patients with Postoperative Cognitive Dysfunction and NSE, S100β Protein Levels Chang | 2018 | Clinical trail | Duplication excluded |
| 195 | WanFangdate | Effect of Electroacupuncture Stimulation on Postoperative Cognitive Disorder of Elderly Patients after General Anesthesia | 2018 | Clinical trail | Duplication excluded |
| 196 | WanFangdate | Research Progress of Traditional Chinese Medicine and Postoperative Cognitive Dysfunction in Elderly Patients | 2018 | Review | Duplication excluded |
| 197 | WanFangdate | Effect of Transcutaneous Electrical Acupoint Stimulation Combined with Dexamethasone on Postoperative Cognitive function in Elderly Patients with Benign Prostatic Hyperplasia | 2018 | Clinical trail | Duplication excluded |
| 198 | WanFangdate | Effect of percutaneous acupoint electrical stimulation on postoperative cognitive function in elderly patients with colorectal cancer | 2017 | Clinical trail | Duplication excluded |
| 199 | WanFangdate | Effect and mechanism of electroacupuncture preconditioning on postoperative cognitive function in aged rats | 2017 | Animal Study | Duplication excluded |
| 200 | WanFangdate | Effect of Electroacupuncture on Cognition and Hippocampal TNF-α and IL-1β Expressions in Rats with Postoperative Cognitive Dysfunction | 2017 | Animal Study | Duplication excluded |
| 201 | WanFangdate | Effects of electroacupuncture on postoperative cognitive dysfunction and AngⅡ/AT1R in the hippocampus in aging rats induced by D-galactose | 2017 | Animal Study | Duplication excluded |
| 202 | WanFangdate | Effects of electrotherapy on postoperative cognitive dysfunction in elderly patients with insulin-resistant joint replacement | 2017 | Clinical trail | Duplication excluded |
| 203 | WanFangdate | Individualized scalp acupuncture for motor dysfunction in stroke: a randomized controlled trial | 2017 | Clinical trail | Duplication excluded |
| 204 | WanFangdate | Effect of Different Frequency Transcutaneous Electrical Acupoint Stimulation Treated on the Postoperative Cognitive Function of Patients undergoing Gynecological Laparoscopy | 2017 | Clinical trail | Duplication excluded |
| 205 | WanFangdate | Effect of Combined Acupuncture and Drug Anesthesia Combined With Electrical Stimulation on Cognitive Function and Serum S-100β Protein in Patients with Abdominal Surgery | 2017 | Clinical trail | Duplication excluded |
| 206 | WanFangdate | Effects of transcutaneous electrical acupoint stimulation on early postop erative cognitive function in patients with transsphenoidal surgery | 2017 | Clinical trail | Duplication excluded |
| 207 | WanFangdate | Effect of electroacupuncture pretreatment on postoperative cognitive function in diabetic rats | 2016 | Animal Study | Duplication excluded |
| 208 | WanFangdate | Effect of Dexmedetomidine Combined Electrical Stimulation on Coanitive Function of Patients Receiving Extracerebral Intervention | 2016 | Clinical trail | Duplication excluded |
| 209 | WanFangdate | TEAS for prevention and treatment of orthodontic toothache and oral dysfunction: a randomized controlled trial | 2016 | Clinical trail | Duplication excluded |
| 210 | WanFangdate | Effect of Transcutaneous Electrical Acupoint Stimulation on Postoperative Cognitive Function in Patients Undergoing Radical Thoracoscopic Lung Cancer Operation | 2016 | Clinical trail | Duplication excluded |
| 211 | WanFangdate | Effects of Transcutaneous Acupoint Electrical Stimulation and Dexmedetomidine on Postoperative Cognitive Dysfunction in Patients Underwent Laparoscopic Surgery | 2016 | Clinical trail | Duplication excluded |
| 212 | WanFangdate | Effect of percutaneous acupoint electrical stimulation on postoperative cognitive function in elderly patients with colorectal cancer | 2016 | Clinical trail | Duplication excluded |
| 213 | WanFangdate | Effects of Methylprednisolone Combined with Transcutaneous AcupiontElectric Stimulation on  Postoperative Cognitive Dysfunction in Elderly Patients | 2016 | Clinical trail | Duplication excluded |
| 214 | WanFangdate | Effect and Mechanism of Acupuncture on Postoperative Cognitive Dysfunction in Aged Rats | 2015 | Animal Study | Duplication excluded |
| 215 | WanFangdate | Effect of TEAS Combined with General Anesthesia on Cognitive Function of Patients Undergoing Gynaecological Laparoscopy | 2015 | Clinical trail | Duplication excluded |
| 216 | WanFangdate | Impacts of electrical acupoint stimulation on postoperative cognitive dysfunction and inflammation  cytokines in elderly patients | 2015 | Clinical trail | Duplication excluded |
| 217 | WanFangdate | Effect of Transcutaneous Acupoint Electrical Stimulation on Postoperative Cognitive Function in Elderly Patients Undergoing Laparoscopic Resection of Rectal Cancer | 2015 | Clinical trail | Duplication excluded |
| 218 | WanFangdate | Effects of electroacupuncture preconditioning on postoperative cognitive dysfunction in elderly patients undergoing joint replacement | 2014 | Clinical trail | Duplication excluded |
| 219 | WanFangdate | Effect of Electroacupuncture on Middle-aged and Elderly POCD Rats’HPA Axis and Cognitive Function | 2014 | Animal Study | Duplication excluded |
| 220 | WanFangdate | Effects of transcutaneous electrical acupoint stimulation combined with inhalation anesthesia on postoperative cognitive function of aged patients with laparoscopic cholecystectomy | 2014 | Clinical trail | Duplication excluded |
| 221 | WanFangdate | Clinical Observation of Electroacupuncture on Postoperative Cognitive Dysfunction in Elderly Patients | 2014 | Clinical trail | Duplication excluded |
| 222 | WanFangdate | Impacts of the different frequencies of electroacupunctrue on cognitive function in patients after abdominal operation under compound anesthesia of acupuncture and drugs | 2013 | Clinical trail | Duplication excluded |
| 223 | WanFangdate | Influences of acupuncture anesthesia on postoperative cognitive dysfunction and S-100βprotein level of the elderly patients of colorectal cancer resection | 2013 | Clinical trail | Duplication excluded |
| 224 | WanFangdate | Effects of Electroacupuncture Assistant General Anesthesia on Postoperative Cognitive Dysfunction of Aged Patients | 2012 | Clinical trail | Duplication excluded |
| 225 | WanFangdate | Effects of Electroacupuncture on Neiguan on postoperative cognitive dysfunction in patients undergoing Off-pump coronary artery bypass grafting | 2011 | Clinical trail | Duplication excluded |
| 226 | WanFangdate | Effect of Controlled Hypotension Combined with Transcutaneous Electrical Acupoint Stimulation on Early Postoperative Cognitive Dysfunction in Spinal Surgery | 2011 | Clinical trail | Duplication excluded |
| 227 | WanFangdate | Effect of Transcutaneous Acupoint Electrical Stimulation on Lipid Peroxidation and Cognitive Function in Patients Experiencing Craniotomy | 2009 | Clinical trail | Duplication excluded |
